# Physical Exercise Promotes a Reduction in Cardiac Fibrosis in the Chronic Indeterminate Form of Experimental Chagas Disease

**DOI:** 10.3389/fimmu.2021.712034

**Published:** 2021-11-04

**Authors:** Yasmin Pedra-Rezende, Juliana M. C. Barbosa, Ana Cristina S. Bombaça, Luiza Dantas-Pereira, Daniel Gibaldi, Glaucia Vilar-Pereira, Hílton Antônio Mata dos Santos, Isalira Peroba Ramos, Natália Lins Silva-Gomes, Otacilio C. Moreira, Joseli Lannes-Vieira, Rubem F. S. Menna-Barreto

**Affiliations:** ^1^ Laboratório de Biologia Celular, Instituto Oswaldo Cruz, Fundação Oswaldo Cruz, Rio de Janeiro, Brazil; ^2^ Laboratório de Biologia das Interações, Instituto Oswaldo Cruz Oswaldo Cruz, Fundação, Rio de Janeiro, Brazil; ^3^ Instituto Brasileiro de Medicina de Reabilitação, Rio de Janeiro, Brazil; ^4^ Faculdade de Farmácia, Universidade Federal do Rio de Janeiro, Rio de Janeiro, Brazil; ^5^ Laboratório de Análise e Desenvolvimento de Inibidores Enzimáticos e Laboratório Multiusuário de Análises por RMN, Universidade Federal do Rio de Janeiro, Rio de Janeiro, Brazil; ^6^ Centro Nacional de Biologia Estrutural e Bioimagem, Universidade Federal do Rio de Janeiro, Rio de Janeiro, Brazil; ^7^ Plataforma de PCR em Tempo Real RPT09A, Laboratório de Biologia Molecular de Doenças Endêmicas, Instituto Oswaldo Cruz, Fundação Oswaldo Cruz, Rio de Janeiro, Brazil

**Keywords:** Chagas disease, experimental model, chronic indeterminate form, physical exercise, cytokines, mitochondrial metabolism, oxidative metabolism, ROS

## Abstract

Chagas disease (CD), caused by the protozoan *Trypanosoma cruzi*, is a neglected tropical disease and a health problem in Latin America. Etiological treatment has limited effectiveness in chronic CD; thus, new therapeutic strategies are required. The practice of physical exercises has been widely advocated to improve the quality of life of CD patients. The most frequent clinical CD manifestation is the chronic indeterminate form (CIF), and the effect of physical exercises on disease progression remains unknown. Here, in a CIF model, we aimed to evaluate the effect of physical exercises on cardiac histological, parasitological, mitochondrial, and oxidative metabolism, electro and echocardiographic profiles, and immunological features. To establish a CIF model, BALB/c and C57BL/6 mice were infected with 100 and 500 trypomastigotes of the Y *T. cruzi* strain. At 120 days postinfection (dpi), all mouse groups showed normal PR and corrected QT intervals and QRS complexes. Compared to BALB/c mice, C57BL/6 mice showed a lower parasitemia peak, mortality rate, and less intense myocarditis. Thus, C57BL/6 mice infected with 500 parasites were used for subsequent analyses. At 120 dpi, a decrease in cardiac mitochondrial oxygen consumption and an increase in reactive oxygen species (ROS) were detected. When we increased the number of analyzed mice, a reduced heart rate and slightly prolonged corrected QT intervals were detected, at 120 and 150 dpi, which were then normalized at 180 dpi, thus characterizing the CIF. Y-infected mice were subjected to an exercise program on a treadmill for 4 weeks (from 150 to 180 dpi), five times per week in a 30–60-min daily training session. At 180 dpi, no alterations were detected in cardiac mitochondrial and oxidative metabolism, which were not affected by physical exercises, although ROS production increased. At 120 and 180 dpi, comparing infected and non-infected mice, no differences were observed in the levels of plasma cytokines, indicating that a crucial biomarker of the systemic inflammatory profile was absent and not affected by exercise. Compared with sedentary mice, trained Y-infected mice showed similar parasite loads and inflammatory cells but reduced cardiac fibrosis. Therefore, our data show that physical exercises promote beneficial changes that may prevent CD progression.

## Introduction

Chagas disease (CD), caused by the protozoan parasite *Trypanosoma cruzi*, is a neglected tropical disease and affects 6 to 7 million people worldwide (1, 2). In the last three decades, rural evasion and migration from endemic Latin American countries to non-endemic countries in North America, Europe, and Asia have changed the epidemiological profile of the disease, increasing the number of individuals at risk of infection (2, 3). CD has two clinical phases: acute and chronic. The acute phase is characterized by patent parasitemia, the presence of inflammatory infiltrates in different tissues and specific IgM synthesis against *T. cruzi* (4). In the chronic phase, approximately 70% of individuals do not show clinical signs or symptoms, characterizing its indeterminate form (CIF) (5, 6). This clinical form is distinguished by the presence of antibodies against *T. cruzi* in the serum, a normal electrocardiogram (ECG) or chest X-ray, and an unaltered esophagus and/or colon radiograph. Most individuals remain in this phase for the rest of their lives (6). Echocardiography (ECHO) and other complementary exams are not common in individuals who present a normal ECG. However, there are CD cases with a normal ECG and changes in other cardiological exams, such as ECHO, myocardial scintigraphy, and magnetic resonance imaging, that are generally mild and not associated with a higher risk of death (7–9). Biopsies from CIF patients have shown discrete or low myocarditis (10, 11). The absence of clinical manifestations in CD may be associated with an individual’s ability to regulate the infection-triggered immune response that controls parasite growth (12). The remaining 30%–40% of patients will develop determinate forms, such as cardiac, digestive, cardio-digestive, and neuronal and/or behavioral disorders (9). Chronic Chagas’ heart disease or chronic chagasic cardiomyopathy (CCC) is the prevalent determined clinical form and the main cause of morbidity and mortality, representing one of the major causes of heart failure (HF) and sudden death in Latin America (8). CCC is characterized by parasite persistence, inflammation, and progressive fibrosis with remodeling of the myocardium and is commonly associated with HF (8). When HF is established, the patient presents a progressive worsening of cardiac functional capacity, which limits daily activities (9). Since there is no specific and effective treatment, CCC is treated similarly to HF of other etiologies (13, 14). Thus, the identification of a specific treatment has become a major challenge. Importantly, the correlation between cardiac inflammation caused by the presence of the parasite added to the intensity of the systemic immune response has been associated with the pathogenesis of CCC (15–17).

*T. cruzi* is an obligate intracellular parasite that is able to infect a wide range of cells and spread throughout tissues. This parasite exhibits a distinct organ tropism, mainly to cardiac muscle cells (18). Due to its biological function, cardiac muscle is rich in mitochondria and highly dependent on oxidative phosphorylation (OXPHOS) for energy production (19, 20). In the last two decades, CCC has been considered a mitochondrial disorder, and morphological alterations such as mitochondrial swelling, irregular membranes, and loss of cristae occur at the beginning of the infection and are exacerbated with the progression and severity of the cardiac form of CD (21, 22). Such mitochondrial dysfunction has been correlated with decreased activity of the respiratory complexes, resulting in inefficient ATP production (23, 24). Despite their classical role in bioenergetics, mitochondria are also responsible for the generation of large amounts of reactive oxygen species (ROS). During mitochondrial respiration, a partial reduction of O_2_ occurs, producing O_2_^-^, H_2_O_2_, or OH^-^ (25). However, ROS production increases exponentially following damage to the electron transport system (25). During *T. cruzi* infection, ROS can be produced due to direct parasite-triggered tissue destruction, the cytotoxicity-mediated immune response, or mitochondrial damage (25). Decreased activity of mitochondrial complexes is involved in the exacerbation of ROS generation in the heart tissue of acute and chronic *T. cruzi*-infected mice (24, 26, 27).

Physical training has been shown to improve the quality of life and to reduce hospitalizations and deaths of patients with heart disease, including CCC (28, 29). Molecularly, physical activity promotes an increase in electron transport system efficiency and mitochondrial biogenesis, as well as clearance of toxic molecules, including ROS and aldehydes (19, 30, 31). Furthermore, physical exercises combined with etiological treatment have been widely supported for use with people affected by CD (32–35). In experimental models of CCC, physical exercises before *T. cruzi* infection have been shown to decrease parasitemia and to improve the immune response and antioxidant defenses in heart tissue (36–38). However, such exacerbations of immune and pro-oxidant responses have been directly associated with CD aggravation (25, 39). In this scenario, anti-inflammatory and antioxidant agents, such as vitamin E and C and curcumin, have been proposed to prove protection against tissue damage (5, 40). In the acute phase of infection in Wistar rats, physical exercises are not recommended since they exacerbate inflammation in cardiac tissue and increase cytokine levels in serum (41). In experimental models of CCC, physical exercises improve morphological and morphometric parameters in heart tissue (42, 43). In patients with CCC, physical exercises ameliorate cutaneous microvascular function, hemodynamics, ECHO, and clinical parameters (33, 34, 44–46). Very little is known about the physiopathology of CIF. In recent years, experimental models have been described attempting to fulfill the lack of knowledge on CIF as well as the mechanisms involved in the pathogenesis and progression of CD (47–49). In the present study, a long-lasting murine model of CIF testing C57BL/6 and BALB/c mice infected with the Y *T. cruzi* strain was initially developed. Next, a CIF model was used to investigate the effects of physical training on the progression of heart disease, thus analyzing parasitological, electrical, and echocardiographic features, systemic cytokine profiles, heart tissue damage, myocarditis, fibrosis, and mitochondrial and oxidative metabolism.

## Material and Methods

### Reagents

Adenosine 5′-diphosphate (ADP), antimycin A (AA), bovine serum albumin (BSA), catalase, cytochrome c from equine hearts, glutathione reduced (GSH), glutathione reductase, malate, β-nicotinamide adenine dinucleotide (NADH), β-nicotinamide adenine dinucleotide 2′-phosphate reduced (NADPH), nitrotetrazolium blue chloride (NBT), paraformaldehyde, pyruvate, rotenone, sodium dithionite, succinic acid, xanthine, and xanthine oxidase were purchased from Sigma-Aldrich (St. Louis, MO, USA). H_2_O_2_ and potassium cyanide were purchased from Merck (Darmstadt, Germany). The CK-MB Liquiform kit was purchased from Labtest (Lagoa Santa, Minas Gerais, Brazil). The BD™ Cytometric Bead Array kit (CBA) was obtained from BD Biosciences (San Jose, CA, USA). OCT Tissue-Tek resin was purchased from Sakura (Torrance, CA, USA). Dihydroetide (DHE) and Pierce™ BCA protein assay kits were purchased from Thermo Fisher (Waltham, MA, USA). GAPDH was obtained from Applied Biosystems (Waltham, MA, USA). The High Pure PCR Template Preparation kit was purchased from Roche (Basel, Switzerland).

### Ethics Statement

Four- to seven-week-old female BALB/c and C57BL/6 mice were obtained from the Institute of Science and Technology in Biomodels (ICTB) of the Oswaldo Cruz Foundation (Fiocruz). All protocols were carried out in accordance with the recommendations of the Guide for the Care and Use of Laboratory Animals of the National Council for Animal Experimentation - COBEA (http://www.cobea.org.br/) and in accordance with federal Law 11.794 (8th of October 2008). The Animal Use Ethics Committee of IOC/Fiocruz (L-002/2019) approved all the procedures used in this study.

### Experimental Model and Infection

Mice were kept under a 12-h light–dark cycle with access to food and water *ad libitum*. Mice were infected intraperitoneally with 100 (Tc 100) or 500 (Tc 500) *T. cruzi* bloodstream trypomastigotes (Y strain, DTU II). These parasites were obtained by cardiac puncture at the peak of parasitemia (7° dpi) in Swiss mice (3 × 10^5^ parasites intraperitoneally route) (50). Parasitemia was estimated using 5 μl of blood obtained from the tail and monitored daily from 5 dpi until the parasitemia was subpatent. Mortality was registered weekly. Animals were randomly chosen for subsequent tests.

### Experimental Groups

For the establishment of a CIF model, BALB/c (H-2^d^) and C57BL/6 (H-2^b^) mice were divided into three groups: non-infected (NI), infected with 100 (Tc 100), or infected with 500 (Tc 500). For subsequent experiments, C57BL/6 mice were divided into four groups: uninfected sedentary (NI S = 14), uninfected trained (NI T = 14), infected sedentary (*Tc* S =14), and infected trained (*Tc* T = 14). Euthanasia was performed by cervical dislocation. Peripheral blood was collected with heparin (2500 UI/ml) by cardiac puncture. The spleen and heart were removed, weighed, sectioned in fragments and stored dry or embedded in OCT Tissue-Tek resin (Sakura, USA), frozen in liquid nitrogen, and stored at -80°C.

### Physical Exercises

At 140 dpi, NI T and *Tc* T mice were subjected to training adaptation on a motorized treadmill (Insight, Brazil) for 1 week (3 days/week), followed by 4 weeks of forced exercise training (5 days/week). During the adaptation period, the mice were subjected to a 4–6-m/min exercise for 15 min. The training protocol consisted of a progressive increase in speed: (a) 6–14 m/min for 30 min/day in the first week; (b) 8–16 m/min for 45 min in the second week; (c) 10–18 m/min for 60 min in the third week; and (d) 20 m/min for 60 min in the fourth week. The treadmill speeds were estimated to provide mice with moderate-intensity exercise, as previously proposed for a *T. cruzi*-infected model of CD (36).

### Muscle Strength Analysis

At 120 and 180 dpi, muscle strength was calculated using a grip strength meter (Grip, FEP 305, Insight, Brazil) as previously described (51, 52). Data are expressed as the mean of the strength intensity = gram-force (gf)/body weight (g) (52).

### Electrocardiographic Analysis

At 120, 150, and 180 dpi, all mice were sedated with diazepam (10 mg/kg), and transducers were placed subcutaneously for derivation (DII) analysis. Traces were recorded using a digital system (Power Lab 20/02) connected to a 2-mV bioamplifier for 1 s (Panlab Instruments, Spain), with records for 2 min. Heart rates (beats per min, bpm), P-wave duration and PR and corrected QT intervals, and QRS complex in milliseconds (ms) were evaluated (53, 54).

### Echocardiographic Analysis

For analysis of cardiac function, mice were anesthetized (inhalation route) with 1.5% isoflurane gas in oxygen with a 1-l/min flow, trichotomized in the precordial region and examined with a Vevo 770 ultrasound device (Visual Sonics, Canada) coupled to a 30-MHz transducer (54, 55) at 180 dpi. The left ventricular ejection fraction (% LVEF), fractional area variation (% FAC), stroke volume, right ventricle area and left ventricle area were determined.

### CK-MB Activity

At 120 and 180 dpi, creatine kinase myocardial MB isoenzyme (CK-MB) activity was measured in plasma using a commercial CK-MB Liquiform kit (Labtest) according to the manufacturer’s recommendations (53).

### Cytokine Analysis

At 120 and 180 dpi, concentrations of the cytokines interleukin-6 (IL-6), interleukin-10 (IL-10), monocyte chemoattractant protein-1 (MCP-1), interferon-γ (IFNγ), tumor necrosis factor (TNF), and interleukin-12p70 (IL-12p70) were evaluated in systemic circulating plasma according to the BD™ Cytometric Bead Array kit.

### Histopathological and Histochemical Analysis

At 120 and 180 dpi, the left ventricles were collected, included in OCT Tissue-Tek resin (Sakura, USA), frozen in liquid nitrogen, and stored at -80°C. To analyze heart inflammation, serial sections (5 μm) were obtained using a CM1850 cryostat (Leica, Germany), fixed in cold acetone, and stained with hematoxylin and eosin (H&E) (56). The quantification of total inflammatory cells was performed by evaluating 100 microscopic fields (magnification, ×400) per mouse in a double-blind fashion (53, 57–59). To evaluate cardiac fibrosis, tissue sections were fixed in a 4% paraformaldehyde solution and stained with *Picrosirius red* (60). The analysis was performed using the ImageJ program, analyzing six photos (magnification, ×100) of each tissue section. The production of ROS in the tissue was evaluated by staining unfixed tissue with 50 μM DHE for 30 min. After staining, the sections were fixed in a 4% paraformaldehyde (PFA) solution. The analysis was performed using the ImageJ program with four photos of each mouse (61).

### Parasite Load in Cardiac Tissue

DNA extraction from cardiac tissue was carried out using the High Pure PCR Template Preparation kit, following the manufacturer’s recommendations. To determine the parasitic load of the cardiac tissue, 5 μl of purified DNA was analyzed by qPCR using the TaqMan system with the Cruzi 1 (5′ASTCGGCTGATCGTTTTCGA3′) and Cruzi 2 (5′AATTCCTCCAAGCAGCGGATA3′) primers, both at 750 nM, and the Cruzi 3 (5′FAM-CA CACACTGGACACCAA-NFQ-MGB3′) TaqMan probe, at 50 nM, specific for the nuclear satellite DNA region of the parasite. As an endogenous internal control in the qPCR aimed at normalizing the parasite load by the quantity of mouse heart tissue, the predesigned TaqMan assay targeting mouse GAPDH (Cat n°. Mm99999915-g) was used. A standard curve was created by spiking 1 × 10^6^ trypomastigotes in 30 mg of non-infected heart tissue, followed by DNA extraction, and generation of a 1:10 serial dilution of DNA in Tris-EDTA buffer, ranging from 10^6^ to 0.1 parasite equivalents. Real-time PCRs were carried out on an Applied Biosystems ViiA 7 Real-Time PCR (Thermo Fisher, USA) thermocycler using the following cycling conditions: 50°C for 2 min, 94°C for 10 min, followed by 40 cycles at 95°C and 58°C for 1 min. Fluorescence was collected after each cycle. All samples were run in duplicate, and the threshold was set at 0.02 for both targets. The parasitic load was reported as parasite equivalents/mg heart tissue (62), and not detected (ND) referred to the absence of parasitism or parasite load below the detection limit of 0.1 parasite equivalents/mg of tissue.

### High-Resolution Respirometry

At 120 and 180 dpi, fresh left ventricle fragments were collected and immersed in ice-cold BIOPS buffer (2.77 mM CaK_2_EGTA, 7.23 mM K_2_EGTA, 20 mM imidazole, 20 mM taurine, 50 mM MES, 0.5 mM DTT, 6, 56 mM MgCl_2_, 5.77 mM ATP, 15 mM phosphocreatine, pH 7.1). Fragments were transferred to BIOPS buffer containing 50 μg/ml saponin and incubated with stirring at 4°C for 30 min. The permeabilized fragments were transferred to Mir05 buffer (0.5 mM EGTA, 3 mM MgCl_2_·6 H_2_O, 60 mM lactobionic acid, 20 mM taurine, 10 mM KH_2_PO_4_, 20 mM HEPES, 110 mM D-sucrose, 1 g/l BSA) for 10 min with stirring at 4°C. Next, the samples were added to 2 ml of Mir05 buffer and analyzed using high-resolution Oxygraphy-2k (Oroboros Co., Innsbruck, Austria) (63). Substrates and inhibitors of the electron transport system were added: 2.5 mM pyruvate + 2 mM malate (substrates of complex I), 2.5 mM ADP (substrate of ATP synthase), 10 mM succinate (substrate of complex II), 10 μM cytochrome c, 0.5 μM rotenone, and 2.5 μM AA. The oxygen flux per mg of tissue was determined using Oroboros DatLab software as described previously (63, 64).

### Enzymatic Activity Assay

Heart tissue fragments were resuspended in phosphate-buffered saline (PBS) with 1% protease inhibitor cocktail and disrupted by sonication. The protein concentration was determined using a Pierce™ BCA protein assay kit. All assays were performed with homogenates. NADH:cytochrome c oxidoreductase (complexes I–III) was monitored by ferricytochrome c reduction at 550 nm (ϵ = 18.5 mM^−1^ cm^−1^) in a reaction mixture containing 50 mM potassium phosphate buffer pH 7.4, 1 mg/ml BSA, 300 μM KCN, 50 μM cytochrome c and 200 μM NADH. The succinate:cytochrome c reductase activity (complexes II–III) was monitored by ferricytochrome c reduction at 550 nm (ϵ = 18.5 mM^−1^ cm^−1^) in the reaction mixture containing 100 mM potassium phosphate buffer pH 7.4, 150 μM equine heart cytochrome c, 3 mM succinic acid, and 1 mM KCN. Next, 10 μM rotenone (complexes I–III) and 2 µM AA (complexes II–III) were added as controls (65). To assess glutathione peroxidase (GPx) activity, the samples were added to the reaction mixture (100 mM potassium phosphate buffer, 200 μM NADPH, 1 mM GSH, 1 U/ml GR, and 300 μM H_2_O_2_), and NADPH reduction was measured at 340 nm (ϵ = 6.22 mM) (66). To assess superoxide dismutase (SOD) activity, the samples were added to the reaction mixture (50 mM potassium phosphate buffer pH 7.4, 0.13 mg/ml BSA, 1 U/ml catalase, 100 μM xanthine, 55 μM NBT and 0.1 U/ml xanthine oxidase), and NBT reduction was measured at 560 nm. For catalase activity, the samples were added to a reaction mixture containing 100 mM potassium phosphate buffer and 100 mM H_2_O_2_, and the decomposition of H_2_O_2_ was measured at 240 nm (ϵ = 39.4 mol^−1^ cm^−1^). All analyses were performed using a SpectraMax M3 Plus 384 spectrophotometer (Molecular Devices, Sunnyvale, USA) (67).

### Statistical Analysis

All data were analyzed using Prism 8 software (GraphPad, San Diego, CA) and expressed as the mean ± standard error of the mean (SEM). The data distribution was evaluated by the Shapiro–Wilk normality test. All statistical tests were performed using the Student’s t-test. One-way test (ANOVA) associated with Tukey’s posttest, two-way (ANOVA) associated with Sidak’s test, and the Mann–Whitney test or the Kruskal–Wallis test associated with Dunn’s posttest. Differences were considered statistically significant when *p* < 0.05.

## Results

### *Trypanosoma cruzi*-Infected BALB/c Mice Show More Prominent Chronic Infection Than C57BL/6 Mice

**Figure 1:**
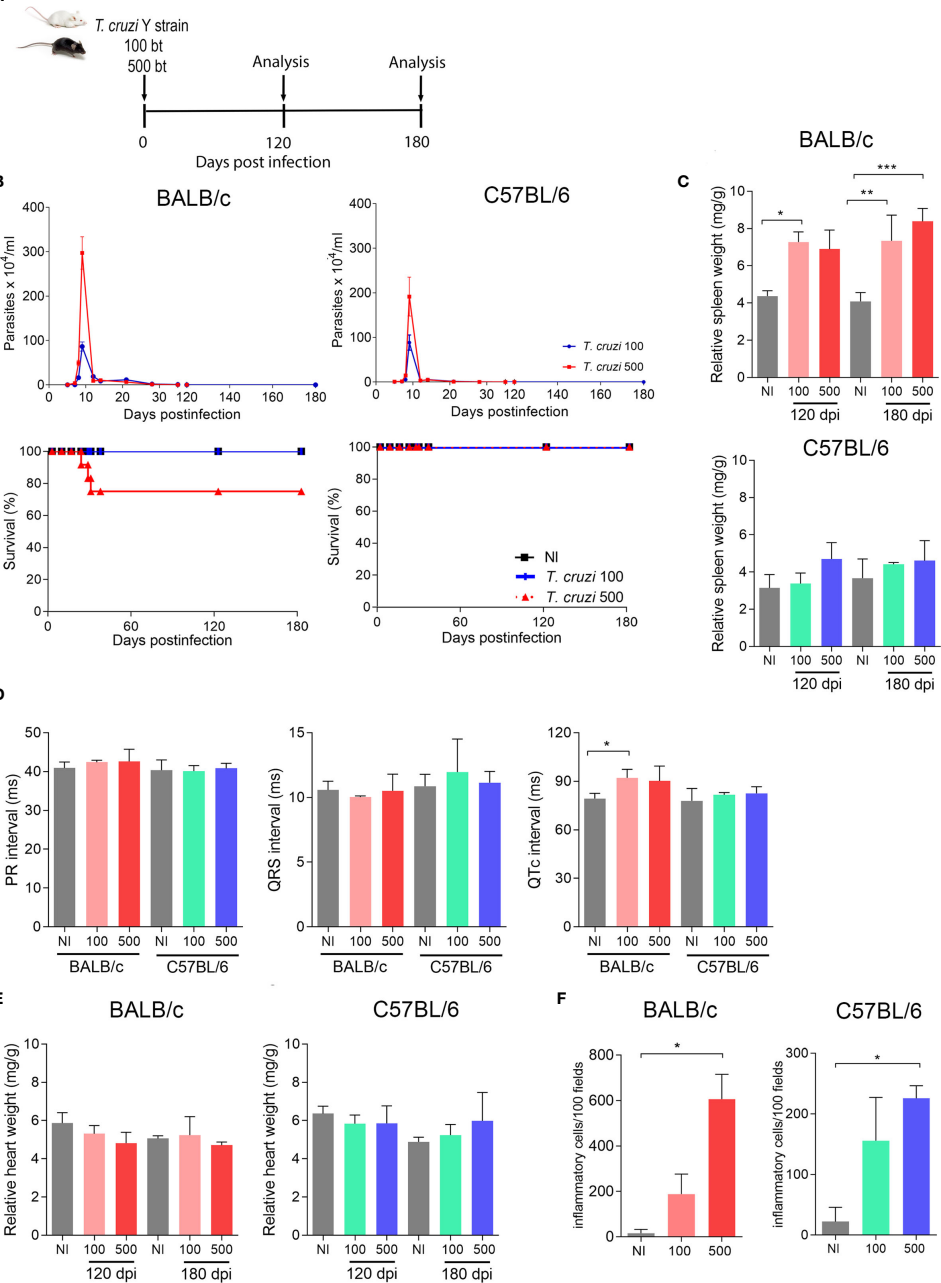
*T. cruzi* infection induces splenomegaly and heart inflammation in Y-infected BALB/c mice. BALB/c and C57BL/6 mice were infected with 100 or 500 bloodstream trypomastigotes (Y strain). **(A)** Experimental design. **(B)** Parasitemia and survival curves. **(C)** Relative spleen weight. **(D)** ECG records showing PR and QTc intervals and the QRS complex (ms). **(E)** Relative heart weight. **(F)** Inflammatory cells in heart tissue at 120 dpi. Data are represented as means ± SEM. Data represent three to four mice per group. Significant differences between infected and uninfected groups. *p < 0.05; **p < 0.01 ***p < 0.001 *(Student’s t-test; Kruskal–Wallis Dunn’s posttest; one-way ANOVA, Tukey posttest)*.

To establish an experimental model of CIF, we used female BALB/c and C57BL/6 mice infected with two low inocula of the Y *T. cruzi* strain and analyzed them at 120 and 180 dpi (**Figure 1A**). With both lineages and inocula, circulating parasites were first detected at 5 dpi, and parasitemia peaked at 9 dpi, when 100% of the mice presented parasites in the peripheral blood. BALB/c mice infected with 500 parasites showed higher parasitemia with the peak in relation to the low inoculum (**Figure 1B**). Furthermore, no differences were observed when comparing parasitemia in C57BL/6 mice that received the two inocula (**Figure 1B**). In both models, parasitemia control occurred between 30 and 35 dpi (**Figure 1B**). In the BALB/c lineage, the inoculum of 500 parasites reduced the survival rate by 33% (3/9), while mice infected with 100 trypomastigotes showed no mortality (0/9) (**Figure 1B**). There was also no mortality in infected C57BL/6 mice up to 180 dpi (0/9) (**Figure 1B**). Female BALB/c mice infected with 100 and 500 parasites showed an increase in the relative spleen weight at 120 and 180 dpi (**Figure 1C**). No difference was detected in the relative spleen weight in infected C57BL/6 mice compared with matched non-infected controls (**Figure 1C**).

At 180 dpi, BALB/c mice infected with 100 parasites showed an increase in the QTc interval. Nevertheless, C57BL/6 mice showed no electrical abnormalities, as exemplified by the absence of prolonged PR and QTc intervals and the QRS complex (**Figure 1D**). At 120 and 180 dpi, both models showed no differences in relative heart weight compared with their age-matched non-infected controls (**Figure 1E**). Analysis of H&E-stained heart sections revealed that BALB/c mice presented a higher number of inflammatory cells when inoculated with 500 parasites than when inoculated with 100 parasites (**Figure 1F**). Nevertheless, infected C57BL/6 mice (100 and 500 parasites) showed a lower number of inflammatory cells scattered in the heart tissue, and only the group infected with 500 parasites showed differences in this parameter compared with the matched non-infected control (**Figure 1F**). Altogether, our data on the survival rate, spleen weight, electrical heart registers, and parasitological and histopathological parameters indicated that C57BL/6 mice showed milder manifestations of acute and chronic *T. cruzi* infection than BALB/c mice. To obtain reproducible data, C57BL/6 mice infected with 500 parasites were chosen as models for subsequent experiments, challenging the effects of physical exercises in mice with CIF of experimental CD.

### Physical Exercise Does Not Affect the Body Weight and Muscle Strength of *Trypanosoma cruzi*-Infected C57BL/6 Mice

**Figure 2:**
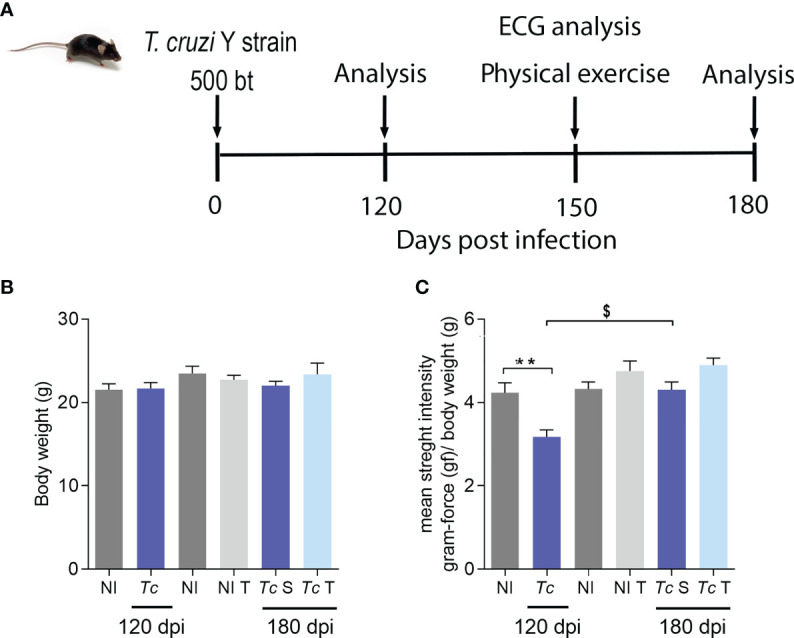
*T. cruzi* infection does not change body weight but reduces muscular strength in C57BL/6 mice. Mice were infected with 500 trypomastigotes (Y strain). **(A)** Experimental design. **(B)** Body weight. **(C)** Muscular strength. The data are represented as means ± SEM. Data represent eight to nine mice per group. Significant differences between infected and uninfected groups. **p < 0.01. Significant differences between infected groups at 120 and 180 dpi. ^$^p > 0.05 (*Test t*; *one-way ANOVA, Tukey posttest; Kruskal–Wallis Dunn’s posttest*; *two-way ANOVA, Sidak’s posttest*).

To assess the effect of physical exercises on clinical aspects in a CIF model of CD, chronically Y-infected C57BL/6 mice were subjected to a training protocol starting at 140 dpi and to physical exercises from 150 to 180 dpi, at which time sedentary (S) and trained (T) mice were compared (**Figure 2A**). Initially, compared with age-matched non-infected controls, body weight was preserved in Y-infected C57BL/6 mice at 120 and 180 dpi, and exercise did not affect whole-body weight (**Figure 2B**). When muscle strength was assessed by the grip strength meter test, Y-infected mice showed a loss of muscle strength at 120 dpi (**Figure 2C**). At 180 dpi, there was no significant difference between sedentary or trained non-infected and *T. cruzi*-infected mice (**Figure 2C**). At 180 dpi compared with 120 dpi, Y-infected mice showed an increase in muscle strength (**Figure 2C**). Nonetheless, comparing only sedentary and trained chronically Y-infected mice, the exercise protocol increased muscle strength (*p* = 0.0334) (**Figure 2C**).

### Physical Exercises Do Not Negatively Affect the Electrical Features of *Trypanosoma cruzi-*Infected C57BL/6 Mice in CIF

**Figure 3:**
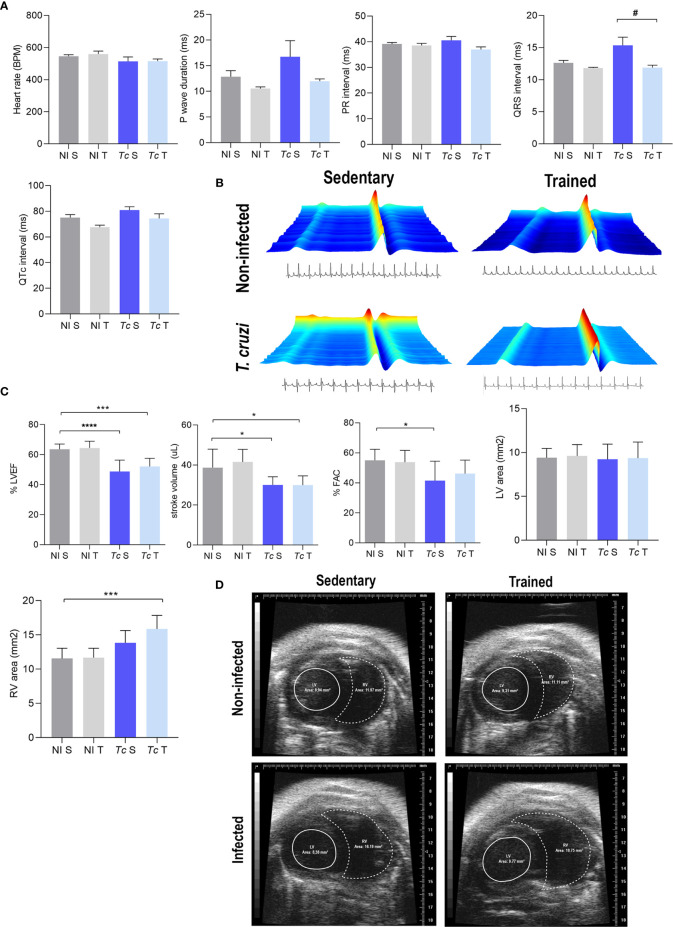
*T. cruzi* infection does not induce electrical alterations but leads to ECHO abnormalities in C57BL/6 mice at 180 dpi. Absence of electrical alterations in C57BL/6-infected mice at 180 dpi. **(A)** ECG records show the average heart rate (beats per minute, bpm), P wave duration (ms), variation in PR and QTc intervals, and QRS complex (ms). **(B)** Representative ECG register segments. **(C)** ECHO records show the % left ventricular ejection fraction (LVEF), stroke volume (µL), % FAC, LV area (mm^2^), and RV area (mm^2^). **(D)** Representative ECHO images of ventricular areas. The data are represented as means ± SEM. Data represent eight to nine mice per group. Significant differences between the untrained and trained groups. ^#^p < 0.05. Significant differences between infected and uninfected groups. *p < 0.05; ***p < 0.001; ****p < 0.0001 (*Kruskal–Wallis Dunn’s posttest*).

The electrocardiographic (ECG) analysis revealed a slight reduction of the heart rate and increased QTc intervals in Y-infected mice in comparison with non-infected mice at 120 dpi (**Supplementary Figure S1A**). At 150 dpi, infected mice showed a slight increase in the QRS complex and QTc interval, with no other ECG changes detected in these mice (**Supplementary Figure S1B**). At 180 dpi, sedentary and trained *T. cruzi*-infected C57BL/6 mice showed no electrical changes compared with matched non-infected mice when heart rate, P wave duration, PR and QTc intervals, and QRS complex were analyzed (**Figures 3A, B**). Furthermore, trained Y-infected mice showed a reduced QRS complex compared with sedentary infected mice. Moreover, the duration of the QRS complex detected in trained infected mice was similar to that found in sedentary and trained non-infected mice at 180 dpi (**Figure 3A**).

### Echocardiographic Changes in *Trypanosoma cruzi*-Infected C57BL/6 Mice at 180 dpi

At 180 dpi, echocardiographic analysis showed that compared with matched non-infected controls, sedentary *T. cruzi*-infected controls showed a decrease in LVEF as well as left ventricle stroke volume and FAC (**Figure 3C**). Physical exercises did not impact these alterations in infected mice (**Figure 3C**). No differences were observed in the LV area in infected mice subjected or not to exercise compared with their matched non-infected controls (**Figures 3C, D**). Conversely, trained Y-infected mice showed an increase in the RV area compared with sedentary non-infected mice, but no differences were detected when sedentary and trained Y-infected mice were compared (**Figures 3C, D**).

### Physical Exercises Do Not Interfere With Heart Weight, Cardiac Parasite Load, Inflammation, and Tissue Integrity

**Figure 4:**
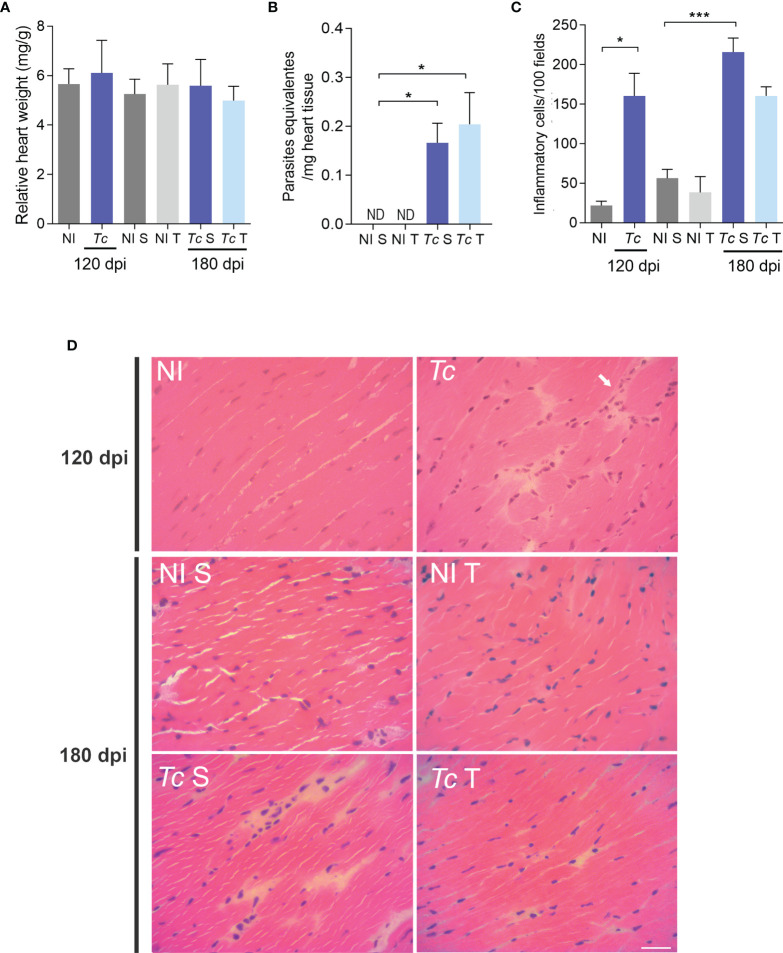
Parasite persistence and inflammation in heart tissue of trained Y-infected mice. **(A)** Relative heart weight. **(B)** Parasite load at 180 dpi. **(C)** Quantitative analysis of inflammatory infiltrates. **(D)** Representative sections stained with H&E (ND = not detected). The data are represented as means ± SEM. Data represent nine mice per group. Significant differences between infected and uninfected groups. *p < 0.05; ***p < 0,001. (*Mann–Whitney; Kruskal–Wallis test*, *Dunn’s posttest*).

Compared with age-matched non-infected mice, no difference was observed in the relative heart weight of Y-infected mice at 120 and 180 dpi. Moreover, physical exercises did not affect this feature (**Figure 4A**). At 180 dpi, the parasite load analyzed by qPCR showed a low parasitic load in the cardiac tissue. In addition, a similar parasite load was detected in the cardiac tissue of sedentary and trained Y-infected mice (**Figure 4B**). At 120 and 180 dpi, Y-infected C57BL/6 mice presented a slight increase in the number of inflammatory cells in the heart tissue compared with non-infected mice. Thus, low-grade scattered inflammatory cell infiltration was observed in the heart tissue of Y-infected C57BL/6 mice (**Figures 4C, D**). Physical exercises did not aggravate this mild inflammation (**Figures 4C, D**). Additionally, analyses of CK-MB activity levels in plasma, a biomarker of cardiomyocyte lesions (53), showed no differences when compared with all experimental groups and at both timepoints (**Supplementary Figure S2A**).

### *Trypanosoma cruzi* Infection and Physical Exercises Do Not Induce Changes in Mitochondrial Respiration and Oxidative Metabolism in Heart Tissue

**Figure 5:**
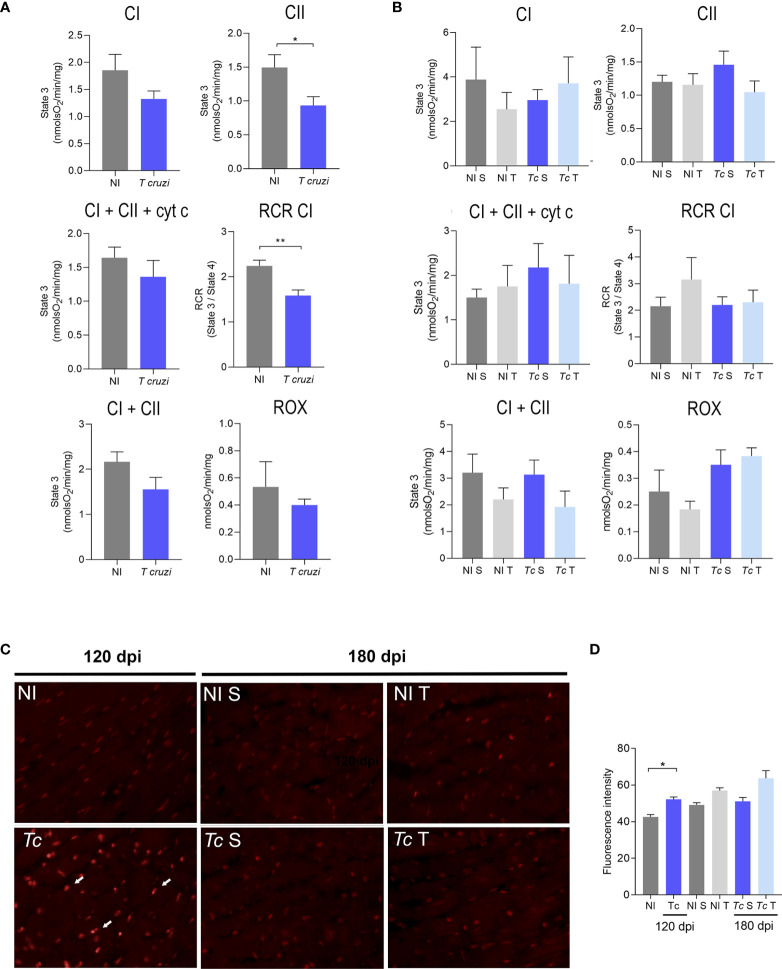
Effects of *T. cruzi* infection and physical exercise on mitochondrial oxygen consumption and ROS production in heart tissue of C57BL/6 mice. **(A, B)** Oxygen consumption at **(A)** 120 dpi and **(B)** 180 dpi. **(C)** Representative sections stained with DHE. **(D)** Densitometric analysis of DHE staining. White arrows indicate DHE staining. The data are represented as means ± SEM. Data represent six to eight mice per group. Significant differences between infected and uninfected groups *p < 0.05; **p < 0.01 (*Mann–Whitney; Kruskal–Wallis test, Dunn’s posttest*).

To assess whether *T. cruzi* infection and physical exercises affect heart mitochondrial metabolism, O_2_ consumption in heart fragments by high-resolution oxygraphy using different substrates was analyzed. At 120 dpi, infected mice showed a decrease in state 3 respiration driven by CII substrate and CI-supported RCR when compared with age-matched non-infected controls (**Figure 5A**). No differences were observed in the other analyzed parameters (**Figure 5A**). At 180 dpi, no significant difference was observed in oxygen consumption in the cardiac tissue in sedentary and trained Y-infected mice (**Figure 5B**). Furthermore, we evaluated the mitochondrial complex activities using a spectrophotometric method, and no differences were observed when comparing sedentary and trained Y-infected mice and their non-infected controls (**Supplementary Figure S2B**). In addition, complementary biochemical analysis showed no significant differences in SOD, GPx, and CAT activities in extracts of cardiac tissue of all analyzed groups (**Supplementary Figure S2B**). DHE analysis pointed to an increase in ROS production in Y-infected mice at 120 dpi (**Figures 5C, D**). At 180 dpi, there was no significant difference in the DHE labeling pattern when comparing sedentary and trained Y-infected controls and their matched non-infected controls (**Figures 5C, D**).

### Physical Exercises Do Not Alter the Cytokine Profile in the CIF Mouse Model

**Figure 6:**
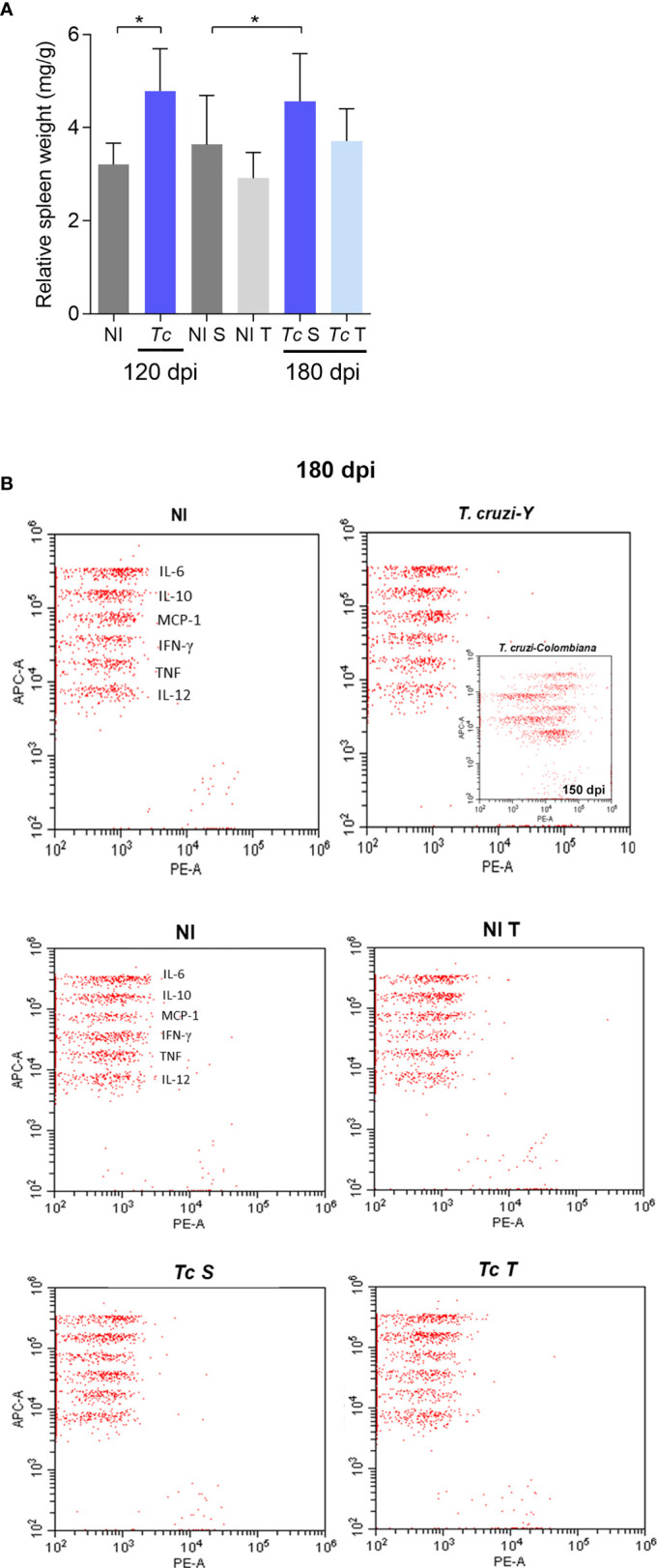
*T. cruzi* infection causes mild splenomegaly but does not induce a systemic inflammatory profile in C57BL/6 mice. **(A)** Relative spleen weight. **(B)** Representative dot plots of Y-infected C57BL/6 mice at 180 dpi and inserted dot plots of Colombian-infected C57BL/6 mice at 150 dpi. The data are represented as means ± SEM. Data represent six to eight mice per group *p < 0,05 (*Mann–Whitney; two-way ANOVA Sidak’s posttest; Kruskal–Wallis test*, *Dunn’s posttest*).

At 120 and 180 dpi, an increase in the relative spleen weight of Y-infected mice was observed, supporting activation of the immune system. Interestingly, in infected mice subjected to physical training, the relative spleen weight was similar to that detected in sedentary and trained non-infected mice (**Figure 6A**). Nonetheless, when comparing only sedentary and trained chronically Y-infected mice, the exercise protocol led to a reduction in spleen weight (*p* = 0.0367). At 120 (data not shown) and 180 dpi, detection of cytokines in systemic circulating plasma by CBA showed no differences in the analyzed cytokines in all groups studied (**Figure 6B**). As a positive control (insert in **Figure 6B**), a strong increase in cytokine expression was detected at 120 dpi in the plasma of C57BL/6 mice infected with the Colombian *T. cruzi* strain, which causes CCC (53). **Table 1** summarizes all data, showing similar concentrations of IL-6, IL-10, MCP-1/CCL2, IFNγ, TNF, and IL-2 in the plasma of the mice in all studied groups.

**Table 1:** Cytokine concentrations in plasma of *Trypanosoma cruzi*-infected C57BL/6.

		IL-12	IL-6	TNF	IFNγ	IL-10	MCP-1
**120 dpi**	**NI**	85.22 ± 24.59	1.61 ± 0.71	29.19 ± 8.23	3.66 ± 0.62	2.90 ± 1.84	49.50 ± 5.86
	***Tc***	79.40 ± 13.39	3.32 ± 1.46	31.87 ± 3.38	3.65 ± 0.69	7.83 ± 3.76	49.64 ± 3.27
**180 dpi**	**NI**	81.70 ± 39.71	0.91 ± 0.58	28.45 ± 13.21	3.20 ± 1.45	11.83 ± 6.34	35.92 ± 11.76
	**NI-T**	78.77 ± 21.24	2.64 ± 0.94	20.03 ± 6.27	3.19 ± 0.65	7.84 ± 4.04	35.06 ± 6.68
	***Tc-*S**	32.74 ± 13.15	1.33 ± 0.60	13.66 ± 4.66	2.51 ± 0.37	5.23 ± 2.69	19.77 ± 6.66
	***Tc-*T**	53.27 ± 23	2.94 ± 0.80	13.27 ± 4.59	2.71 ± 0.89	20.03 ± 8.13	24.96 ± 7.23

### Physical Exercises Lead to a Decrease in Collagen Deposition in Cardiac Tissue in the CIF Mouse Model

**Figure 7:**
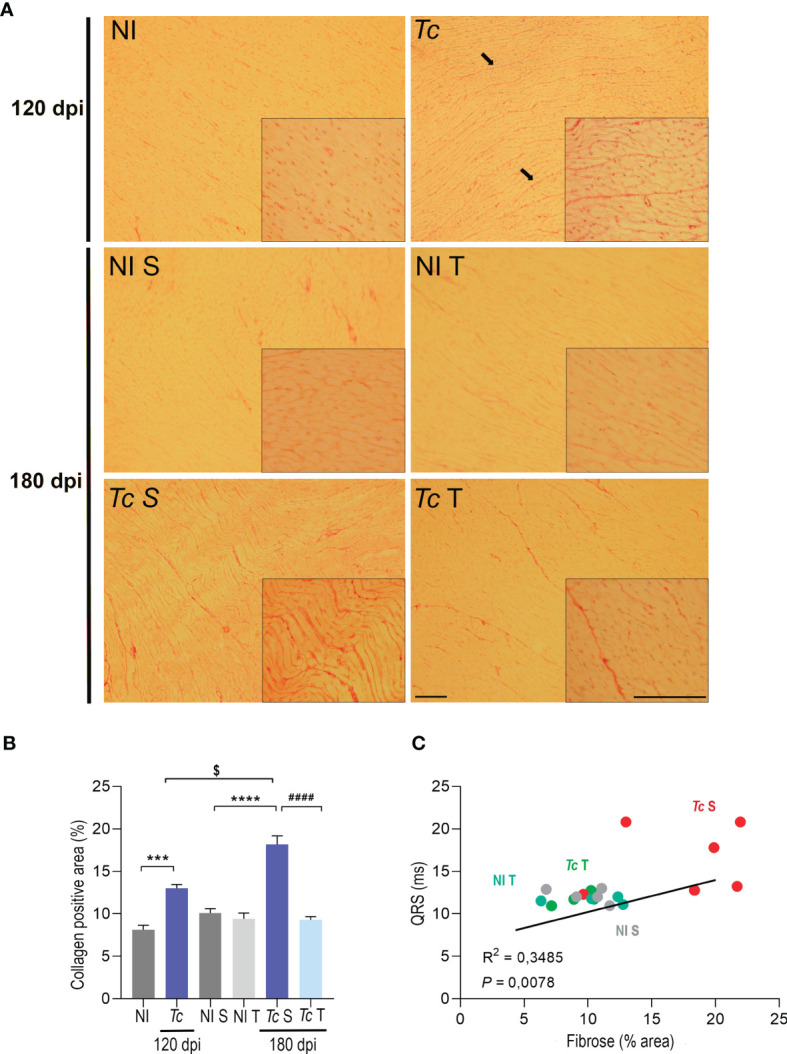
Physical exercise induce a reduction in collagen deposition in the hearts of C57BL/6-infected mice. **(A)** Representative sections stained with *Picrosirius red.* Black arrows indicate positive staining for *Picrosirius red*.** (B)** Densitometric analysis of *Picrosirius red* staining. **(C)** Correlations of the QTc interval and collagen deposition. The data are represented as means ± SEM. Data represent six to eight mice per group. Significant differences between infected and uninfected groups. ***p < 0.001 and ****p < 0.0001; significant differences between the untrained and trained groups, ^####^p < 0.0001; significant differences comparing infected groups at 120 dpi and 180 dpi ^$^p < 0.0001 (Mann–Whitney; *two-way ANOVA, Sidak’s posttest; Kruskal–Wallis* test, *Dunn’s posttest*).

Specific staining of histopathological heart sections was used to assess collagen deposition. At 120 and 180 dpi, increased collagen deposition was detected in the heart tissue of sedentary infected mice (**Figure 7A**). Analysis of the collagen-stained area showed that when compared with their age-matched non-infected controls, Y-infected mice presented increased deposition of collagen of 1.5- and 3.6-fold at 120 and 180 dpi, respectively, thus supporting the progression of fibrosis (**Figure 7B**). At 180 dpi, trained Y-infected mice showed a remarkable decrease in collagen deposition in comparison to sedentary mice (**Figures 7A, B**). Interestingly, a positive correlation between the QRS complex and cardiac fibrosis was observed. Further, trained Y-infected mice developed collagen deposition and the QRS complex similar to sedentary and trained non-infected mice (**Figure 7C**), thus supporting that more than hampering progression of the increase in the QRS complex, physical exercises promoted reversion of fibrosis.

## Discussion

In this study, we established an experimental model of chronic *T. cruzi* infection that reproduces aspects of the CIF of CD. Next, we used this model to evaluate the effects of physical exercise on the progression of CD, analyzing cardiac parasitological and histopathological parameters, mitochondrial and oxidative metabolism, electro and echocardiographic profiles, and immunological aspects. Experimental models that reproduce CD clinical forms and pathophysiological aspects are crucial to comprehending the biological and molecular mechanisms of the disease and to testing new therapeutic strategies. Previous studies have described CIF models (47–49, 68, 69); however, these studies did not confirm whether the infected mice exhibited critical aspects of CIF. Therefore, we aimed to develop an experimental model of long-term chronic infection that represents features of CIF patients, as described previously (9, 70, 71), thereby carrying out analyses of clinical, parasitological, inflammatory, electrical and immunological aspects using a unique approach.

Female BALB/c and C57BL/6 mice, representing susceptible and resistant lineages to *T. cruzi* infection, respectively, were employed to establish a CIF model (72). Females are more resistant than males to infection by *T. cruzi*, resulting in the survival of female to the long-term chronic phase of infection. Previous studies have shown that the participation of hormones such as estradiol could play a relevant role in the vulnerability of female mice, as compared with males, to *T. cruzi* infection (72–75). Here, both mouse lineages infected with two low inocula (100 and 500 trypomastigotes) reproduced features of the CD chronic phase, such as subpatent parasitemia. In long-term infection, BALB/c mice showed higher inflammation than C57BL/6 mice, as explained by the higher susceptibility of BALB/c mice to infection. Indeed, BALB/c mice present a Th2-enriched immune response profile, while C57BL/6 mice display a Th1-enriched response, with different abilities to deal with intracellular parasite infection, as Th1-driven immunity is related to resistance to *T. cruzi* infection (76). BALB/c mice developed more severe parasitological and histopathological manifestations of chronic *T. cruzi* infection than C57BL/6 mice. The initial ECG analysis showed that infected C57BL/6 mice showed no alterations in the long-term infection, therefore reproducing aspects present in the CIF of CD (9). Additionally, in all groups of chronically Y-infected mice, the relative heart weights found resembled those of their age-matched non-infected controls, contrasting with the relative enlargement of the heart detected in murine models of CCC (53, 59). Spleen weight was increased only in the infected BALB/c mice at both time points analyzed. Thus, considering aspects such as the results obtained and the reproducibility of the data, the C57BL/6 lineage and the inocula of 500 parasites were chosen to test our hypotheses using a model that reproduces aspects of the CIF of CD.

Previous studies have shown that C57BL/6 mice have reduced performance during forced physical exercise on a treadmill and better voluntary wheel-running performance than BALB/c mice (77). Nonetheless, C57BL/6 mice showed good performance during forced exercise training on a motorized treadmill, which resulted in an improvement in cardiac fibrosis. The initial analysis of physical exercises on aspects of the CIF model showed no alterations in the overall body weight and a trend toward improved muscle strength in non-infected and infected mice. Patients with chronic HF subjected to combined aerobic and resistance exercise training presented improved muscular strength but no change in body weight (78). Clinical evaluation revealed a reduced heart rate and increased in the QRS complex and QTc interval at 120 and 150 dpi. Importantly, no significant ECG alterations were observed at 180 dpi. As infection progressed, trained Y-infected mice presented a reduced QRS complex compared with Y-infected sedentary mice, resembling the non-infected group. Nonetheless, at the endpoint, sedentary or trained Y-infected mice presented similar ECHO alterations, such as a reduced LVEF, stroke volume, and FAC and an increased RV area. According to the II Brazilian Consensus on Chagas Disease 2015, CIF patients presented a positive serology and/or positive parasitological exams for *T. cruzi*. However, ECG or chest-X-ray and esophagus and/or colon radiographs showed no alterations. As long as the ECG records are unaltered, other routine complementary exams, such as ECHO, are not recommended (8, 9). Based on this classification, Y-infected mice would be included in the CIF; however, the presence of ECHO alterations would lead to exclusion of this group (9). The importance of ECHO investigation for the clinical classification of CD patients has been evaluated previously, showing segmental lesions and systolic dysfunction in 1% of patients with normal ECG results (7), reinforcing the importance of a complete cardiac evaluation of chronic CD patients. Considering the CIF models described previously, our data suggest that ECHO is a demanding exam for better characterization of CIF in experimental models. Thus, T. *cruzi*-infected C57BL/6 mice present aspects of CIF patients who do not show ECG variations but ECHO alterations in long-term chronic infection. Thus, adoption of our model may allow us to explore different aspects of CIF and to study the pathophysiology and progression of CD as well as the effects of physical exercises on these features. Surprisingly, similar to sedentary mice, the trained Y-infected mice showed a decrease in LVEF and stroke volume. These findings were not expected, since physical exercises related to improving cardiovascular function are well established, such as LVEF progression, even in patients who present reductions of this parameter (34, 78–83). However, a study examining the effects of exercises on the LVEF of patients with HF showed no difference in this parameter (84). Furthermore, we showed that physical exercises promoted an increase in the RV area in long-term Y-infected mice. Non-infected Wistar rats subjected to moderate physical exercises showed RV hypertrophy, characterized by an increase in capillary density, which improves the diffusion and transport of oxygen in the tissue and, consequently, ameliorates cardiac function (85). Based on our findings, it is not possible to affirm whether physical activity influences CD progression. Thus, we conclude that a study of this nature needs to be carried out with a large number of mice together with follow-up analyses for a long period, which we hope to address in the near future.

The program of physical exercises used did not affect the relative heart weight, which remained similar to the age-matched non-infected controls and contrasted with the heart enlargement observed in a model of CCC in C57BL/6 mice (59). Regardless of the chronic CD form, the persistence of parasites and/or their antigens in cardiac tissue has been well described in humans and experimental models (10, 53, 62). Here, a low cardiac parasitic load was similarly detected in sedentary or trained Y-infected mice at 180 dpi. Therefore, physical exercises did not alter the control of cardiac parasitism. The intensity of myocarditis has been shown to be directly correlated with the level of parasite antigens in the heart during the chronic phase of CD (10). In addition, there is a consensus that CD is a multifactorial disease, with *T. cruzi* persistence and unregulated immune responses playing crucial roles in CCC onset and progression (16). Therefore, our data support the conclusion that the adoption of physical exercises in CIF individuals is not expected to contribute to increased heart parasitism. During CIF, low-grade myocarditis has been described (15, 17), which was corroborated in our model. Our data revealed the presence of a small number of inflammatory cells scattered in the cardiac tissue in the long-term infection of C57BL/6 mice. Previous models of mild and severe long-term CCC have shown that the intensity of myocarditis is associated with the severity of heart parasitism and cardiomyocyte lesions (58). Hence, we evaluated whether long-term *T. cruzi* infection and physical exercises affect cardiomyocytes, thus assessing CK-MB activity, an important non-invasive marker of cardiomyocyte injury in acute and chronic phases of an experimental CD model (51, 57, 59). Importantly, no damage was observed in cardiomyocytes in the long-term infection (at 120 dpi) and in sedentary or trained Y-infected C57BL/6 mice (at 180 dpi), which indicated that the low-grade inflammation and parasitism detected in our model were not sufficient to cause significant tissue damage. Furthermore, physical exercises did not affect tissue integrity in mice with CIF.

To assess whether *T. cruzi* infection and physical exercises affect cardiac tissue mitochondrial energy metabolism, oxygen consumption was analyzed by high-resolution respirometry (63). Our data showed that *T. cruzi* infection induced a reduction in oxygen consumption in chronic infection (at 120 dpi). The addition of Cit c did not increase oxygen consumption in the cardiac tissue of mice infected or not, thereby confirming the integrity of the mitochondrial membranes (64). Surprisingly, a reduction in respiratory complex activity was not observed. The alterations observed in mitochondrial respiration were discreet and were only detected using a high-resolution technique. Colorimetric analysis of enzymatic activities is less sensitive. Previous studies have shown that the decrease in CI activity is more evident during the acute phase of *T. cruzi* infection, with the activities of CII and CIII being constitutively inhibited in the heart during the acute and chronic phases of infection (20, 22). In addition, it has been observed that ADP-stimulated respiration and ATP synthesis sustained by pyruvate/malate and succinate substrates decrease significantly in cardiac tissue and in isolated mitochondria from infected mice (20, 64), corroborated by our data. In long-term CIF (at 180 dpi), cardiac tissue mitochondrial OXPHOS was not affected. A previous study carried out in experimental CIF models using Swiss mice infected with Tulaheun and SGO-Z12 strains showed dysfunction in citrate synthase activity and respiratory complexes, as well as mitochondrial matrix and cristae disorganization in heart tissue (47). However, in the present study, only parasitic load and histopathological and morphological analyses were performed, which we considered insufficient to confirm CIF. Furthermore, other studies using experimental CIF models have shown modifications of cardiac contractility, alterations of some components of the cardiac β adrenergic system, and ECG alterations (48, 49, 86). Theoretically, the CIF of CD is a stage of host–parasite equilibrium that may explain the apparent clinical silence (4). The different structural and functional changes in the mitochondria of the cardiac tissue may be related to the host genetic background and/or the infecting parasite strain, among other physiopathological conditions. Therefore, further assays will be required to clarify the existence of minor damage in cardiac mitochondria in our CIF model. Our data indicate that the program of forced physical exercises to which our experimental CIF mice were subjected was safe and did not cause mitochondrial damage detectable by the respirometry and enzymatic activity assays employed herein.

In *T. cruzi* infection, ROS are produced as a consequence of tissue damage caused by the parasite, cytotoxic reactions mediated by the immune system and secondary mitochondrial damage (25). It has been reported that the mitochondrial membrane potential is impaired by *T. cruzi* invasion, which results in inefficient electron transport chain activity and increased ROS production in cardiomyocytes (25). Due to the reduction in oxygen consumption, electron leakage from the respiratory chain was expected at 120 dpi as a result of increased superoxide formation and oxidative stress in the heart tissue of *T. cruzi*-infected mice, and we observed an increase in ROS production. The impairment of the mitochondrial electron transport system in the myocardium caused by *T. cruzi* infection, as evidenced by the decrease in oxygen uptake and reduction in the activity of the complexes, can lead to an increase in ROS generation (20, 27). Ultrastructural analysis of myocardial necropsies from patients and infected mice with heart abnormalities have shown mitochondrial alterations such as irregular membranes and loss of cristae (21, 87).

As previously mentioned, physical exercises have been implemented as a complementary therapeutic strategy in CCC patients (33, 44). In cardiovascular diseases, physical exercises induce cardioprotective effects, increase endogenous antioxidant defenses, and modulate the immune response (81). The modulation of inflammatory processes and oxidative state by exercises can be an interesting approach to prevent CD progression (37, 40). In this scenario, we hypothesized that physical exercise in CIF individuals could promote beneficial changes in cardiac tissue, minimizing possible CD evolution. Such a hypothesis is especially important due to a lack of information in low-income populations that are commonly subjected to economic activities requiring physical effort (e.g., farmers, stevedores, construction workers). Therefore, our work supports that physical exercise promotes beneficial changes in heart tissue, suggesting that this intervention may prevent cardiac alterations induced by *T. cruzi* infection. Here, we showed that in long-term Y infection (at 180 dpi), physical exercises did not promote any change in OXPHOS and ROS production. An increase in ROS levels during physical exercises and during *T. cruzi* infection has been well established in isolated events (88, 89). Injuries induced by oxidative stress are a common finding in the chagasic myocardium (25). Increased systemic TNF and NO serum levels have been correlated with reductions in GPx and SOD levels and proposed to affect the pathology of chronically infected patients (90). During the acute phase, physical exercises increase ROS and RNS generation as well as the activity of antioxidant enzymes in the cardiac muscle tissue that are not sufficient to decrease tissue damage (41). Conversely, physical exercises administered before infection promote an increase in ROS and RNS and the activity of antioxidant enzymes that limit tissue damage in skeletal muscle tissue (61). In our study, physical exercises did not increase ROS or antioxidant defenses in cardiac tissue. Plasma cytokine levels are considered an excellent marker of the systemic inflammatory profile (70). It has been shown that individuals with CIF present a balance between inflammatory and anti-inflammatory cytokines, while individuals with CCC show a predominant inflammatory response (15, 67, 91, 92). In experimental models of mild and severe CCC, TNF serum levels are related to the severity of cardiac inflammation, cardiomyocyte lesions, and fibrosis (58). However, CIF patients show no differences in TNF, IFNγ, and IL-2 levels when compared with healthy individuals (70, 92). Furthermore, similar IFNγ and IL-10 serum levels have been detected in CIF patients and healthy individuals (93), as corroborated by our data. Thus, our findings support that Y-infected C57BL/6 mice present a profile of serum cytokines that is compatible with that of CIF patients, and physical exercises do not alter this immunological condition. The splenomegaly observed in our CIF model was not sufficient to cause an increase in systemic inflammation. Furthermore, the splenomegaly observed in our model was mild compared with that observed in a murine model of CC described in previous studies by our group. In the model of CCC using C57BL/6 mice infected with the Colombian strain of *T. cruzi* (51), we observed a spleen enlargement of approximately four-fold compared with non-infected mice, while in our model, spleen enlargement was approximately 1.6-fold.

Diffuse myocarditis with destruction of the cardiac fibers and replacement by fibrosis has been described in CCC patients (15). In addition, an increase in fibronectin deposition has been observed in the hearts of CCC models, which was more evident in the severe CCC model in C3H/He mice than in the mild CCC model in C57BL/6 mice (58). Indeed, CCC is considered a progressive fibrotic disorder (15). Although the presence of fibrosis in the myocardium is a hallmark of CCC, some studies have described fibrosis in CIF patients at similar levels to that observed in the cardiac form of CD without LV dysfunction (71, 94). Here, Y-infected mice presented an increase in collagen deposition, which might be correlated with the observed ECHO alterations. The progressive accumulation of collagen has been shown to reduce the efficiency of the regulatory mechanism of muscular contraction (95). Nevertheless, we observed that physical exercises induced a reduction in collagen deposition in the heart tissue of trained Y-infected C57BL/6 mice. Y-infected Wistar rats subjected to a preinfection treadmill running program have shown a decrease in cardiac fibrosis (37), a feature corroborated by our findings when long-term infected mice were subjected to physical exercises. Although no electrical changes were observed in Y-infected mice, some mice showed an increase in the QRS complex and in the mean value. Exercises promoted the maintenance of the QRS complex at 180 dpi, similar to non-infected mice. The increase in the QRS complex duration has been previously associated with the severity of fibrosis and the reduction in the LVEF fraction in CCC patients (96). In this context, our data showed a correlation between decreased collagen deposition and QRS complex duration in trained Y-infected mice. Thus, the significant decrease in collagen deposition suggests that physical exercises prevent possible cardiac damage related to the infection *via* a mechanism that remains to be determined.

In our CIF model, we demonstrated that intervention using physical exercises did not affect mitochondrial and oxidative metabolism, cardiac parasite load or inflammation, or the systemic cytokine profile. Physical exercises promote beneficial effects, such as the improvement of fibrosis, thus suggesting that this intervention could prevent possible alterations induced by *T. cruzi* infection and thereby contribute to the prevention of disease progression.

## Data Availability Statement

All datasets generated for this study are included in the article/**Supplementary Material**.

## Ethics Statement

The animal study was reviewed and approved by the Animal Use Ethics Committee of IOC/Fiocruz (L-002/2019).

## Author Contributions

Conceived and designed the experiments: JL-V, RM-B, and YP-R. Performed the experiments: JL-V, RM-B, YP-R, JB, AB, LD-P, DG, GV-P, HS, IR, and NS-G. Analyzed the data: JL-V, RM-B, YP-R, GV-P, HS, IR, and OM. Wrote the manuscript: JL-V, RM-B, and YP-R. All authors contributed to the article and approved the submitted version.

## Funding

This work was supported by grants from Fundação Carlos Chagas Filho de Amparo à Pesquisa do Estado do Rio de Janeiro/FAPERJ (E-26/202.572/2019, E-26/210.190/2018) and the Brazilian Research Council/CNPq (BPP 306037/2019-0). J. Lannes-Vieira and RS Menna-Barreto are research fellows of the Brazilian Research Council/CNPq and CNE or JCNE/Fundação Carlos Chagas Filho de Amparo à Pesquisa do Estado do Rio de Janeiro/FAPERJ. This study was financed in part by the “Coordenação de Aperfeiçoamento de Pessoal de Nível Superior do Brasil” (CAPES) – Finance Code 001.

## Conflict of Interest

The authors declare that the research was conducted in the absence of any commercial or financial relationships that could be construed as a potential conflict of interest.

The reviewer LC declared a shared affiliation with several of the authors, HS and IR, to the handling editor at the time of review.

## Publisher’s Note

All claims expressed in this article are solely those of the authors and do not necessarily represent those of their affiliated organizations, or those of the publisher, the editors and the reviewers. Any product that may be evaluated in this article, or claim that may be made by its manufacturer, is not guaranteed or endorsed by the publisher.

## Supplementary Material

Supplementary Figure S1
*T. cruzi*-infected C57BL/6 mice presented electrical abnormalities at 120 and 150 dpi. **(A)** At 120 dpi. **(B)** At 150 dpi. ECG records showed the average heart rate (beats per minute, bpm), P wave duration (ms), variation in PR and QTc intervals, and QRS complex (ms). The data are represented as means ± SEM. Data represent eight to nine mice per group. Significant differences between infected and uninfected groups *P < 0.05 (*Mann–Whitney* test).

Supplementary Figure S2 Effect of *T. cruzi* infection and physical exercise on CK-MB, mitochondrial and antioxidant activities in C57BL/6 mice. **(A)** CKMB activity. **(B)** Mitochondrial complex and antioxidant activities. Data are represented as means ± SEM. Data represent six to eight mice per group (*Mann–Whitney*; *Kruskal–Wallis test*, *Dunn’s posttest*).

## References

[1] Chagas C. Nova Tripanozomiaze Humana: Estudos Sobre a Morfolojia E O Ciclo Evolutivo do Schizotrypanum Cruzi N. Gen., N. Sp., Ajente Etiolojico De Nova Entidade Morbida do Homem. Mem Inst Oswaldo Cruz (1909) 1:159–218. doi: 10.1590/S0074-02761909000200008

[2] World Health Organization. Chagas Disease (Also Known as American Trypanosomiasis) (2021). World Health Organization. Available at: https://www.who.int/news-room/fact-sheets/detail/chagas-disease-(american-trypanosomiasis) (Accessed Acessed January, 2021).

[3] Coura JR Viñas PA. Chagas Disease: A New Worldwide Challenge. Nature (2010) 465:S6–7. doi: 10.1038/nature09221 20571554

[4] Andrade DV Gollob KJ Dutra WO. Acute Chagas Disease: New Global Challenges for an Old Neglected Disease. PLoS Negl Trop Dis (2014) 8:1–10. doi: 10.1371/journal.pntd.0003010 PMC4117453 25077613

[5] Novaes RD Sartini MVP Rodrigues JPF Gonçalves RV Santos EC Souza RLM. Curcumin Enhances the Anti-Trypanosoma Cruzi Activity of Benznidazole-Based Chemotherapy in Acute Experimental Chagas Disease. Antimicrob Agents Chemother (2016) 60(6):3355–64. doi: 10.1128/AAC.00343-16 PMC4879395 27001816

[6] Macêdo V. Indeterminate Form of Chagas Disease. Mem Inst Oswaldo Cruz (1999) 94:311–6. doi: 10.1590/S0074-02761999000700059 10677745

[7] Viotti RJ Vigliano C Laucella S Lococo B Petti M Bertocchi G. Value of Echocardiography for Diagnosis and Prognosis of Chronic Chagas Disease Cardiomyopathy Without Heart Failure Heart (2004) 90(6):655–60. doi: 10.1136/hrt.2003.018960 PMC1768261 15145872

[8] Rassi A Rassi A Marin-Neto JA. Chagas Disease. Lancet (2010) 375:1388 1402. doi: 10.1016/S0140-6736(10)60061-X 20399979

[9] Dias JCP Ramos AN Gontijo ED Luquetti A Shikanai-Yasuda MA Coura JR. 2nd Brazilian Consensus on Chagas Disease, 2015. Rev Soc Bras Med Trop (2016) 49(Suppl 1):3–60. doi: 10.1590/0037-8682-0505-2016 27982292

[10] Higuchi ML De Brito T Martins Reis M Barbosa A Bellotti G Pereira-Barreto AC. Correlation Between Trypanosoma Cruzi Parasitism and Myocardial Inflammatory Infiltrate in Human Chronic Chagasic Myocarditis: Light Microscopy and Immunohistochemical Findings. Cardiovasc Pathol (1993) 2:101–6. doi: 10.1016/1054-8807(93)90021-S 25990604

[11] Reis MM Higuchi MDL Benvenuti LA Aiello VD Gutierrez PS Bellotti G. An *in Situ* Quantitative Immunohistochemical Study of Cytokines and IL-2R+ in Chronic Human Chagasic Myocarditis: Correlation With the Presence of Myocardial Trypanosoma Cruzi Antigens. Clin Immunol Immunopathol (1997) 83:165–72. doi: 10.1006/clin.1997.4335 9143377

[12] Brener Z Gazzinelli RT. Immnunological Control of Trypanosoma Cruzi Infection and Pathogenesis of Chagas’ Disease. Int Arch Allergy Immunol (1997) 114:103–10. doi: 10.1159/000237653 9338602

[13] Rassi A de Rezende JM Luquetti AO Rassi A. Clinical Phases and Forms of Chagas Disease. 1st ed. Amsterdam: Elsevier Inc (2010). doi: 10.1016/B978-0-12-384876-5.00027-7

[14] Rassi A Marin-Neto JA Rassi A. Chronic Chagas Cardiomyopathy: A Review of the Main Pathogenic Mechanisms and the Efficacy of Aetiological Treatment Following the BENznidazole Evaluation for Interrupting Trypanosomiasis (BENEFIT) Trial. Mem Inst Oswaldo Cruz (2017) 112:224–35. doi: 10.1590/0074-02760160334 PMC5319366 28225900

[15] Higuchi MDL Benvenuti LA Reis MM Metzger M. Pathophysiology of the Heart in Chagas’ Disease: Current Status and New Developments. Cardiovasc Res (2003) 60:96–107. doi: 10.1016/S0008-6363(03)00361-4 14522411

[16] Lannes-Vieira J Silverio JC Pereira IR Vinagre NF Carvalho CME Paiva CN. Chronic Trypanosoma Cruzi-Elicited Cardiomyopathy: From the Discovery to the Proposal of Rational Therapeutic Interventions Targeting Cell Adhesion Molecules and Chemokine Receptors - How to Make a Dream Come True. Mem Inst Oswaldo Cruz (2009) 104:226–35. doi: 10.1590/S0074-02762009000900029 19753478

[17] Simões MV Romano MMD Schmidt A Martins KSM Marin-Neto JA. Chagas Disease Cardiomyopathy. Int J Cardiovasc Sci (2018) 31:173–89. doi: 10.5935/2359-4802.20180011

[18] Kayama H Takeda K. The Innate Immune Response to Trypanosoma Cruzi Infection. Microbes Infect (2010) 12:511–7. doi: 10.1016/j.micinf.2010.03.005 20348008

[19] Fulghum K Hill BG. Metabolic Mechanisms of Exercise-Induced Cardiac Remodeling. Front Cardiovasc Med (2018) 5:127. doi: 10.3389/fcvm.2018.00127 30255026 PMC6141631

[20] Wen JJ Garg NJ. Manganese Superoxide Dismutase Deficiency Exacerbates the Mitochondrial ROS Production and Oxidative Damage in Chagas Disease. PLoS Negl Trop Dis (2018) 12:1–15. doi: 10.1371/journal.pntd.0006687 PMC6078326 30044789

[21] Garg N Popov VL Papaconstantinou J. Profiling Gene Transcription Reveals a Deficiency of Mitochondrial Oxidative Phosphorylation in Trypanosoma Cruzi-Infected Murine Hearts: Implications in Chagasic Myocarditis Development. Biochim Biophys Acta - Mol Basis Dis (2003) 1638:106–20. doi: 10.1016/S0925-4439(03)00060-7 12853116

[22] Báez A Lo Presti MS Rivarola HW Mentesana GG Pons P Fretes R. Mitochondrial Involvement in Chronic Chagasic Cardiomyopathy. Trans R Soc Trop Med Hyg (2011) 105:239–46. doi: 10.1016/j.trstmh.2011.01.007 21470646

[23] Uyemura SA. Energetics of Heart Mitochondria During Acute Phase of Trypanosoma Cruzi Infection in Rats. Int J Biochem Cell Biol (1995) 27:1183–9. doi: 10.1016/1357-2725(95)00073-X 7584604

[24] Vyatkina G Bhatia V Gerstner A Papaconstantinou J Garg N. Impaired Mitochondrial Respiratory Chain and Bioenergetics During Chagasic Cardiomyopathy Development. Biochim Biophys Acta - Mol Basis Dis (2004) 1689:162–73. doi: 10.1016/j.bbadis.2004.03.005 15196597

[25] Gupta S Wen JJ Garg NJ. Oxidative Stress in Chagas Disease. Interdiscip Perspect Infect Dis (2009) 2009:1–8. doi: 10.1155/2009/190354 PMC2696642 19547716

[26] Wen JJ Bhatia V Popov VL Garg NJ. Phenyl-α-Tert-Butyl Nitrone Reverses Mitochondrial Decay in Acute Chagas’ Disease. Am J Pathol (2006) 169:1953–64. doi: 10.2353/ajpath.2006.060475 PMC1762476 17148660

[27] Wen JJ Yachelini PC Sembaj A Manzur RE Garg NJ. Increased Oxidative Stress is Correlated With Mitochondrial Dysfunction in Chagasic Patients. Free Radic Biol Med (2006) 41:270–6. doi: 10.1016/j.freeradbiomed.2006.04.009 16814107

[28] Davies EJ Moxham T Rees K Singh S Coats AJ Ebrahim S. Exercise Based Rehabilitation for Heart Failure. Cochrane Database Syst Rev (2010) 2014(4):CD003331. doi: 10.1002/14651858.cd003331.pub3 20393935

[29] Belardinelli R Georgiou D Cianci G Purcaro A. 10-Year Exercise Training in Chronic Heart Failure: A Randomized Controlled Trial. J Am Coll Cardiol (2012) 60:1521–8. doi: 10.1016/j.jacc.2012.06.036 22999730

[30] Ascensão A Ferreira R Magalhães J. Exercise-Induced Cardioprotection - Biochemical, Morphological and Functional Evidence in Whole Tissue and Isolated Mitochondria. Int J Cardiol (2007) 117:16–30. doi: 10.1016/j.ijcard.2006.04.076 16860886

[31] Moreira JBN Wohlwend M Fenk S Åmellem I Flatberg A Kraljevic J. Exercise Reveals Proline Dehydrogenase as a Potential Target in Heart Failure. Prog Cardiovasc Dis (2019) 62:193–202. doi: 10.1016/j.pcad.2019.03.002 30867130

[32] Fialho PH Tura BR De Sousa AS De Oliveira CR Cristiane C Soares S. Effects of an Exercise Program on the Functional Capacity of Patients With Chronic Chagas ‘ Heart Disease , Evaluated by Cardiopulmonary Testing. Rev Soc Bras Med Trop (2012) 45:220–4. doi: 10.1590/S0037-86822012000200016 22534996

[33] Nascimento BR Lima MMO Nunes MCP de Alencar MCN Costa HS Pinto Filho MM. Effects of Exercise Training on Heart Rate Variability in Chagas Heart Disease. Arq Bras Cardiol (2014) 103:201–8. doi: 10.5935/abc.20140108 PMC4193067 25098373

[34] Mediano MFF Mendes FSNS Pinto VLM Da Silva GMS Da Silva PS Carneiro FM. Cardiac Rehabilitation Program in Patients With Chagas Heart Failure: A Single-Arm Pilot Study. Rev Soc Bras Med Trop (2016) 49:319–28. doi: 10.1590/0037-8682-0083-2016 27384829

[35] Alves RL Cardoso BRL Ramos IPR Oliveira BS dos Santos ML de Miranda AS. Physical Training Improves Exercise Tolerance, Cardiac Function and Promotes Changes in Neurotrophins Levels in Chagasic Mice. Life Sci (2019) 232:116629. doi: 10.1016/j.lfs.2019.116629 31276687

[36] Schebeleski-Soares C Occhi-Soares RC Franzói-de-Moraes SM de Oliveira DMM Almeida FN de Ornelas Toledo MJ. Preinfection Aerobic Treadmill Training Improves Resistance Against Trypanosoma Cruzi Infection in Mice. Appl Physiol Nutr Metab (2009) 34:659–65. doi: 10.1139/H09-053 19767801

[37] Novaes RD Gonçalves RV Penitente AR Bozi LHM Neves CA Maldonado IRSC. Modulation of Inflammatory and Oxidative Status by Exercise Attenuates Cardiac Morphofunctional Remodeling in Experimental Chagas Cardiomyopathy. Life Sci (2016) 152:210–9. doi: 10.1016/j.lfs.2016.03.053 27040670

[38] Lucchetti BFC Zanluqui NG Raquel HA Lovo-Martins MI Tatakihara VLH Belém MO. Moderate Treadmill Exercise Training Improves Cardiovascular and Nitrergic Response and Resistance to Trypanosoma Cruzi Infection in Mice. Front Physiol (2017) 8:315. doi: 10.3389/fphys.2017.00315 28572772 PMC5435761

[39] Paiva CN Feijó DF Dutra FF Carneiro VC Freitas GB Alves LS. Oxidative Stress Fuels Trypanosoma Cruzi Infection in Mice. J Clin Invest (2012) 122:2531–42. doi: 10.1172/JCI58525DS1 PMC3386808 22728935

[40] Maçao LB Filho DW Pedrosa RC Pereira A Backes P Torres MA. Antioxidant Therapy Attenuates Oxidative Stress in Chronic Cardiopathy Associated With Chagas’ Disease. Int J Cardiol (2007) 123:43–9. doi: 10.1016/j.ijcard.2006.11.118 17328977

[41] Mendonça AAS Gonçalves RV Souza-Silva TG Maldonado IRSC Talvani A Natali AJ. Concomitant Exercise Training Attenuates the Cardioprotective Effects of Pharmacological Therapy in a Murine Model of Acute Infectious Myocarditis. Life Sci (2019) 230:141–9. doi: 10.1016/j.lfs.2019.05.059 31129142

[42] Preto E Lima NEA Simardi L Fonseca FLA Filho AAF Maifrino LBM. Effect of Mild Aerobic Training on the Myocardium of Mice With Chronic Chagas Disease. Biol Targets Ther (2015) 9:87–92. doi: 10.2147/BTT.S85283 PMC4590415 26445527

[43] Ferraboli R Ornelas EDM Fonseca FLA Veiga GL Cardoso CG Marques MR. Effect of Mild Aerobic Exercise in Atrial Granules of Mice With Chronic Chagas Disease. Int J Cardiovasc Sci (2018) 31:585–93. doi: 10.5935/2359-4802.20180060

[44] Lima MMO Rocha MOC Nunes MCP Sousa L Costa HS Alencar MCN. A Randomized Trial of the Effects of Exercise Training in Chagas Cardiomyopathy. Eur J Heart Fail (2010) 12:866–73. doi: 10.1093/eurjhf/hfq123 20675669

[45] Mediano MFF Mendes FSNS Pinto VLM da Silva PS Hasslocher-Moreno AM de Sousa AS. Reassessment of Quality of Life Domains in Patients With Compensated Chagas Heart Failure After Participating in a Cardiac Rehabilitation Program. Rev Soc Bras Med Trop (2017) 50:404–7. doi: 10.1590/0037-8682-0429-2016 28700063

[46] Mendes FSNS Mediano MFF de Castro FCS da Silva PS Carneiro FM de Holanda MT. Effect of Physical Exercise Training in Patients With Chagas Heart Disease (From the PEACH STUDY). Am J Cardiol (2020) 125(9):1413–20. doi: 10.1016/j.amjcard.2020.01.035 32171439

[47] Báez AL Lo Presti MS Fretes R Díaz C Pons P Bazán PC. Chronic Indeterminate Phase of Chagas’ Disease: Mitochondrial Involvement in Infection With Two Strains. Parasitology (2013) 140:414–21. doi: 10.1017/S0031182012001771 23137884

[48] Bustamante JM Rivarola HW Fernández AR Enders JE Fretes R Palma JA. Indeterminate Chagas’ Disease: Trypanosoma Cruzi Strain and Re-Infection are Factors Involved in the Progression of Cardiopathy. Clin Sci (2003) 104:415–20. doi: 10.1042/CS20020245 12653687

[49] Lo Presti MS Rivarola HW Bustamante JM Fernández AR Enders JE Levin G. Some Components of the Cardiac β-Adrenergic System are Altered in the Chronic Indeterminate Form of Experimental Trypanosoma Cruzi Infection. Int J Parasitol (2008) 38:1481–92. doi: 10.1016/j.ijpara.2008.04.009 18582889

[50] De Oliveira GM Diniz RL Batista W Batista MM Correa CB De Araújo-Jorge TC. Fas Ligand-Dependent Inflammatory Regulation in Acute Myocarditis Induced by Trypanosoma Cruzi Infection. Am J Pathol (2007) 171:79–86. doi: 10.2353/ajpath.2007.060643 17591955 PMC1941608

[51] Pereira IR Vilar-Pereira G Moreira OC Ramos IP Gibaldi D Britto C. Pentoxifylline Reverses Chronic Experimental Chagasic Cardiomyopathy in Association With Repositioning of Abnormal CD8+ T-Cell Response. PLoS Negl Trop Dis (2015) 9:1–23. doi: 10.1371/journal.pntd.0003659 PMC4366205 25789471

[52] Vilar-Pereira G Castaño Barrios L da Silva AA Martins Batista A Resende Pereira I Moreira OC. Memory Impairment in Chronic Experimental Chagas Disease: Benznidazole Therapy Reversed Cognitive Deficit in Association With Reduction of Parasite Load and Oxidative Stress in the Nervous Tissue. PLoS One (2021) 16:e0244710. doi: 10.1371/journal.pone.0244710 33400707 PMC7785227

[53] Silverio J Pereira IR Cipitelli MC Vinagre NF Rodrigues MM Gazzinelli RT. CD8+ T-Cells Expressing Interferon Gamma or Perforin Play Antagonistic Roles in Heart Injury in Experimental Trypanosoma Cruzi-Elicited Cardiomyopathy. PLoS Pathog (2012) 8(4):e1002645. doi: 10.1371/journal.ppat.1002645 22532799 PMC3330123

[54] Vilar-Pereira G Carneiro VC Mata-Santos H Vicentino ARR Ramos IP Giarola NLL. Resveratrol Reverses Functional Chagas Heart Disease in Mice. PLoS Pathog (2016) 12:1–19. doi: 10.1371/journal.ppat.1005947 PMC5082855 27788262

[55] Ferreira RR Abreu RS Vilar-Pereira G Degrave W Meuser-Batista M Ferreira NVC. TGF-β Inhibitor Therapy Decreases Fibrosis and Stimulates Cardiac Improvement in a Pre-Clinical Study of Chronic Chagas’ Heart Disease. PLoS Negl Trop Dis (2019) 13:1–27. doi: 10.1371/journal.pntd.0007602 PMC6690554 31365537

[56] Chen TH Liu SW Chen MR Cho KH Chen TY Chu PH. Neonatal Death and Heart Failure in Mouse With Transgenic HSP60 Expression. BioMed Res Int (2015) 2015:539805. doi: 10.1155/2015/539805 26504810 PMC4609373

[57] Pereira IR Vilar-Pereira G Silva AA Moreira OC Britto C Sarmento EDM. Tumor Necrosis Factor is a Therapeutic Target for Immunological Unbalance and Cardiac Abnormalities in Chronic Experimental Chagas’ Heart Disease. Mediators Inflammation (2014) 2014:798078. doi: 10.1155/2014/798078 PMC4130030 25140115

[58] Pereira IR Vilar-Pereira G da Silva AA Lannes-Vieira J. Severity of Chronic Experimental Chagas’ Heart Disease Parallels Tumour Necrosis Factor and Nitric Oxide Levels in the Serum: Models of Mild and Severe Disease. Mem Inst Oswaldo Cruz (2014) 109:289–98. doi: 10.1590/0074-0276140033 PMC4131780 24937048

[59] Gibaldi D Vilar-Pereira G Pereira IR Silva AA Barrios LC Ramos IP. CCL3/Macrophage Inflammatory Protein-1α Is Dually Involved in Parasite Persistence and Induction of a TNF- and Ifnγ-Enriched Inflammatory Milieu in Trypanosoma Cruzi-Induced Chronic Cardiomyopathy. Front Immunol (2020) 11:306. doi: 10.3389/fimmu.2020.00306 32194558 PMC7063958

[60] Lewis MD Francisco AF Jayawardhana S Langston H Taylor MC Kelly JM. Imaging the Development of Chronic Chagas Disease After Oral Transmission. Sci Rep (2018) 8:1–8. doi: 10.1038/s41598-018-29564-7 30050153 PMC6062536

[61] Novaes RD Gonçalves RV Penitente AR Cupertino MC Maldonado IRSC Talvani A. Parasite Control and Skeletal Myositis in Trypanosoma Cruzi-Infected and Exercised Rats. Acta Trop (2017) 170:8–15. doi: 10.1016/j.actatropica.2017.02.012 28223068

[62] Vilar-Pereira G Pereira IR De Souza LA Moreira OC Da Silva AA Britto C. Combination Chemotherapy With Suboptimal Doses of Benznidazole and Pentoxifylline Sustains Partial Reversion of Experimental Chagas’ Heart Disease. Antimicrob Agents Chemother (2016) 60:4297–309. doi: 10.1128/AAC.02123-15 PMC4914640 27161638

[63] Pesta D Gnaiger E. High-Resolution Respirometry: OXPHOS Protocols for Human Cells and Permeabilized Fibers From Small Biopsies of Human Muscle. Methods Mol Biol (2012) . 2012:810. doi: 10.1007/978-1-61779-382-0_3 22057559

[64] Wen JJ Yin YW Garg NJ. PARP1 Depletion Improves Mitochondrial and Heart Function in Chagas Disease: Effects on POLG Dependent mtDNA Maintenance. PLoS Pathog (2018) 14:1–24. doi: 10.1371/journal.ppat.1007065 PMC5979003 29851986

[65] Spinazzi M Casarin A Pertegato V Salviati L Angelini C. Assessment of Mitochondrial Respiratory Chain Enzymatic Activities on Tissues and Cultured Cells. Nat Protoc (2012) 7:1235–46. doi: 10.1038/nprot.2012.058 22653162

[66] Flohé L Günzler WA. Assays of Glutathione Peroxidase. Methods Enzymol (1984) 105:114–20. doi: 10.1016/S0076-6879(84)05015-1 6727659

[67] Weydert C Cullen J. Measurement of Superoxide Dismutase, Catalase, and Glutathione Peroxidase in Cultured Cells and Tissue. Nat Protocol (2011) 5(1):51–66. doi: 10.1038/nprot.2009.197 PMC2830880 20057381

[68] Bazán PC Lo Presti MS Rivarola HW Triquell MF Fretes R Fernández AR. Chemotherapy of Chronic Indeterminate Chagas Disease: A Novel Approach to Treatment. Parasitol Res (2008) 103:663–9. doi: 10.1007/s00436-008-1029-x 18512075

[69] Scarim CB de Andrade CR da Rosa JA dos Santos JL Chin CM. Hydroxymethylnitrofurazone Treatment in Indeterminate Form of Chronic Chagas Disease: Reduced Intensity of Tissue Parasitism and Inflammation—A Histopathological Study. Int J Exp Pathol (2018) 99:236–48. doi: 10.1111/iep.12289 PMC6302791 30320480

[70] Pérez AR Silva-Barbosa SD Roggero E Calmon-Hamaty F Villar SR Gutierrez FR. Immunoendocrinology of the Thymus in Chagas Disease. Neuroimmunomodulation (2011) 18:328–38. doi: 10.1159/000329494 21952685

[71] Noya-Rabelo MM Macedo CT Larocca T Machado A Pacheco T Torreão J. The Presence and Extension of Myocardial Fibrosis in the Undetermined Form of Chagas’ Disease: A Study Using Magnetic Resonance. Arq Bras Cardiol (2018) 110:124–31. doi: 10.5935/abc.20180016 PMC5855905 29466491

[72] De Souza EM Rivera M Araújo-Jorge TC De Castro SL. Modulation Induced by Estradiol in the Acute Phase of Trypanosoma Cruzi Infection in Mice. Parasitol Res (2001) 87:513–20. doi: 10.1007/s004360100376 11484845

[73] Mena-Marín AL Zeledón R Morales JA Pereira M Urbina A. Influencia Del Sexo En La Susceptibilidad De Ratones Swiss a Trypanosoma Cruzi. Bol Malariol y Salud Ambient (2012) 52:233–44.

[74] Guedes-Da-Silva FH Batista DGJ Da Silva CF Meuser MB Simões-Silva MR De Araújo JS. Different Therapeutic Outcomes of Benznidazole and VNI Treatments in Different Genders in Mouse Experimental Models of Trypanosoma Cruzi Infection. Antimicrob Agents Chemother (2015) 59:7564–70. doi: 10.1128/AAC.01294-15 PMC4649169 26416857

[75] Santos CD Levy AMA Toldo MPA Azevedo AP Prado Júnior JC. Haematological and Histopathological Findings After Ovariectomy in Trypanosoma Cruzi Infected Mice. Vet Parasitol (2007) 143:222–8. doi: 10.1016/j.vetpar.2006.08.038 17081692

[76] Bryan MA Guyach SE Norris KA. Specific Humoral Immunity Versus Polyclonal B Cell Activation in Trypanosoma Cruzi Infection of Susceptible and Resistant Mice. PLoS Negl Trop Dis (2010) 4(7):e733. doi: 10.1371/journal.pntd.0000733 20625554 PMC2897841

[77] Lerman I Harrison BC Freeman K Hewett TE Allen DL Robbins J. Genetic Variability in Forced and Voluntary Endurance Exercise Performance in Seven Inbred Mouse Strains. J Appl Physiol (1985) . 2002 92(6):2245–55. doi: 10.1152/japplphysiol.01045.2001 12015333

[78] Maiorana A O’Driscoll G Cheetham C Collis J Goodman C Rankin S. Combined Aerobic and Resistance Exercise Training Improves Functional Capacity and Strength in CHF. J Appl Physiol (2000) 88(5):1565–70. doi: 10.1152/jappl.2000.88.5.1565 10797113

[79] Ellingsen O Halle M Conraads V Stoylen A Dalen H Delagardelle C. High-Intensity Interval Training in Patients With Heart Failure With Reduced Ejection Fraction. Circulation (2017) 135:839–49. doi: 10.1161/CIRCULATIONAHA.116.022924 PMC5325251 28082387

[80] Zhang Y Cao HX Jiang P Tang HQ. Cardiac Rehabilitation in Acute Myocardial Infarction Patients After Percutaneous Coronary Intervention. Med (2018) 97(8):e9785. doi: 10.1097/MD.0000000000009785 PMC5841979 29465559

[81] Pinckard K Baskin KK Stanford KI. Effects of Exercise to Improve Cardiovascular Health. Front Cardiovasc Med (2019) 6:69. doi: 10.3389/fcvm.2019.00069 31214598 PMC6557987

[82] Passino C Severino S Poletti R Piepoli MF Mammini C Clerico A. Aerobic Training Decreases B-Type Natriuretic Peptide Expression and Adrenergic Activation in Patients With Heart Failure. J Am Coll Cardiol (2006) 47(9):1835–9. doi: 10.1016/j.jacc.2005.12.050 16682309

[83] Chen YM Li ZB Zhu M Cao YM. Effects of Exercise Training on Left Ventricular Remodelling in Heart Failure Patients: An Updated Meta-Analysis of Randomised Controlled Trials. Int J Clin Pract (2012) 66(8):782–91. doi: 10.1111/j.1742-1241.2012.02942.x 22805270

[84] Hambrecht R Gielen S Linke A Fiehn E Yu J Walther C. Effects of Exercise Training on Left Ventricular Function and Peripheral Resistance in Patients With Chronic Heart Failure: A Randomized Trial. J Am Med Assoc (2000) 283:3095–101. doi: 10.1001/jama.283.23.3095 10865304

[85] Anversa P Levicky V Beghi C McDonald SL Kikkawa Y. Morphometry of Exercise-Induced Right Ventricular Hypertrophy in the Rat. Circ Res (1983) 52:57–64. doi: 10.1161/01.RES.52.1.57 6848210

[86] Enders JE Paglini P Fernandez AR Marco F Palma JA. Cardiac Beta-Receptors in Experimental Chagas’ Disease. Rev Inst Med Trop Sao Paulo (1995) 37(1):59–62. doi: 10.1590/s0036-46651995000100009 7569641

[87] Parada H Carrasco HA Añez N Fuenmayor C Inglessis I. Cardiac Involvement is a Constant Finding in Acute Chagas’ Disease: A Clinical, Parasitological and Histopathological Study. Int J Cardiol (1997) 60:49–54. doi: 10.1016/S0167-5273(97)02952-5 9209939

[88] Kawamura T Muraoka I. Exercise-Induced Oxidative Stress and the Effects of Antioxidant Intake From a Physiological Viewpoint. Antioxidants (2018) 7(9):119. doi: 10.3390/antiox7090119 PMC6162669 30189660

[89] Paiva CN Medei E Bozza MT. ROS and Trypanosoma Cruzi: Fuel to Infection, Poison to the Heart. PLoS Pathog (2018) 14:1–19. doi: 10.1371/journal.ppat.1006928 PMC5908069 29672619

[90] Pérez-Fuentes R Guégan JF Barnabé C López-Colombo A Salgado-Rosas H Torres-Rasgado E. Severity of Chronic Chagas Disease is Associated With Cytokine/Antioxidant Imbalance in Chronically Infected Individuals. Int J Parasitol (2003) 33:293–9. doi: 10.1016/S0020-7519(02)00283-7 12670514

[91] Bilate AMB Cunha-Neto E. Chagas Disease Cardiomyopathy: Current Concepts of an Old Disease. Rev Inst Med Trop Sao Paulo (2008) 50:67–74. doi: 10.1590/S0036-46652008000200001 18488083

[92] Chaves AT de Assis JSGE Araújo Fiuza J Carvalho AT Ferreira KS Fares RCG. Immunoregulatory Mechanisms in Chagas Disease: Modulation of Apoptosis in T-Cell Mediated Immune Responses. BMC Infect Dis (2016) 16:1–11. doi: 10.1186/s12879-016-1523-1 27138039 PMC4852404

[93] D’Ávila DA Guedes PMM Castro AM Gontijo ED Chiari E Galvão LMC. Immunological Imbalance Between IFN-γ and IL-10 Levels in the Sera of Patients With the Cardiac Form of Chagas Disease. Mem Inst Oswaldo Cruz (2009) 104:100–5. doi: 10.1590/S0074-02762009000100015 19274383

[94] Rochitte CE Oliveira PF Andrade JM Ianni BM Parga JR Ávila LF. Myocardial Delayed Enhancement by Magnetic Resonance Imaging in Patients With Chagas’ Disease: A Marker of Disease Severity. J Am Coll Cardiol (2005) 46:1553–8. doi: 10.1016/j.jacc.2005.06.067 16226184

[95] Higuchi ML Fukasawa S De Brito T Parzianello LC Bellotti G Ramires JAF. Different Microcirculatory and Interstitial Matrix Patterns in Idiopathic Dilated Cardiomyopathy and Chagas’ Disease: A Three Dimensional Confocal Microscopy Study. Heart (1999) 82(3):279–85. doi: 10.1136/hrt.82.3.279 PMC1729160 10455076

[96] Strauss DG Cardoso S Lima JAC Rochitte CE Wu KC. ECG Scar Quantification Correlates With Cardiac Magnetic Resonance Scar Size and Prognostic Factors in Chagas’ Disease. Heart (2011) 97(5):357–61. doi: 10.1136/hrt.2010.21004 21245474

