# Supplementary figures and images for: Physical Exercise Promotes a Reduction in Cardiac Fibrosis in the Chronic Indeterminate Form of Experimental Chagas Disease
Source: Front Immunol. 2021 Nov 4;12:712034. doi: 10.3389/fimmu.2021.712034 (PMC8599157; doi:10.3389/fimmu.2021.712034)

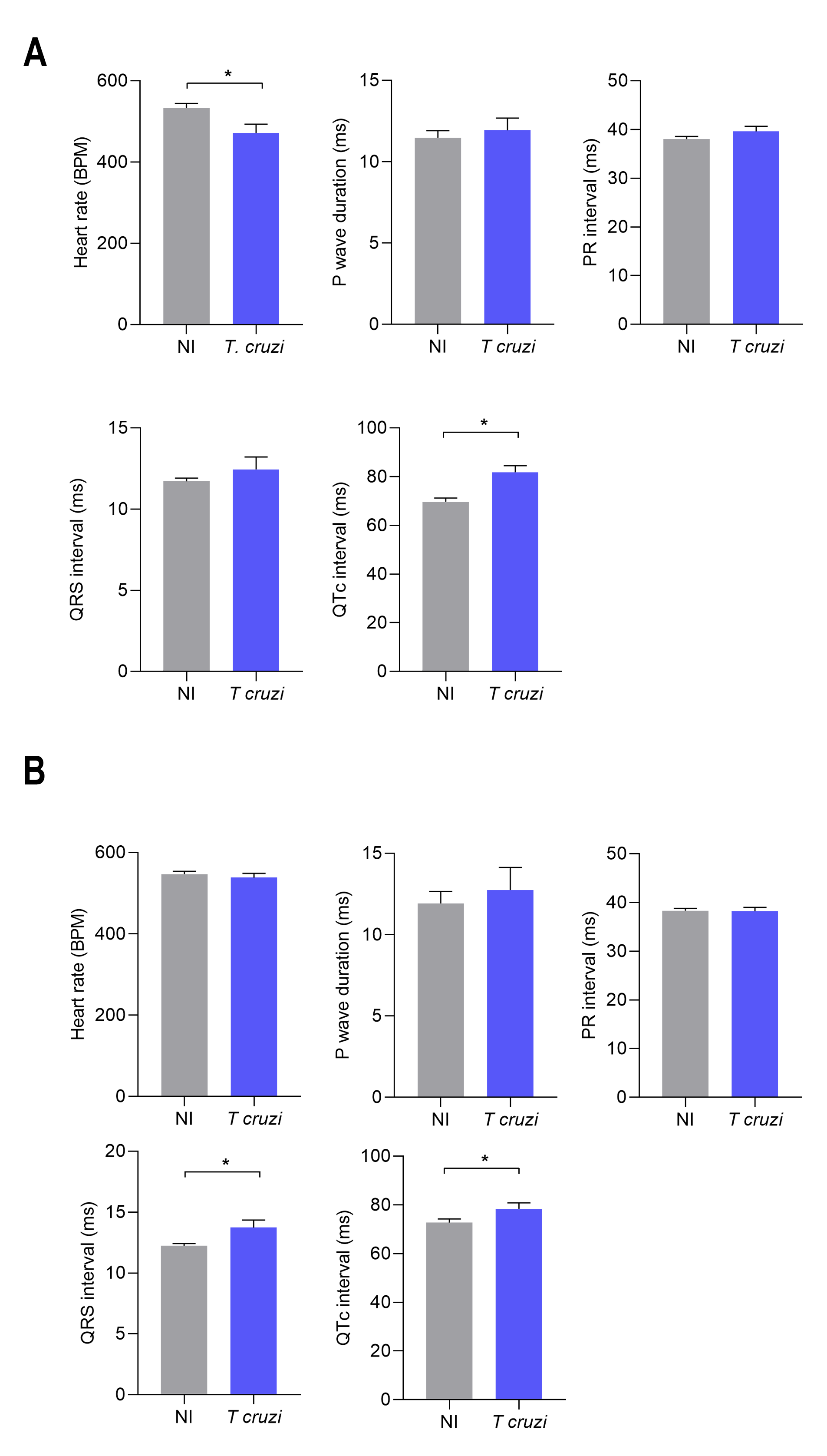

Supplement: Supplementary Figure S1 — T. cruzi-infected C57BL/6 mice presented electrical abnormalities at 120 and 150 dpi. (A) At 120 dpi. (B) At 150 dpi. ECG records showed the average heart rate (beats per minute, bpm), P wave duration (ms), variation in PR and QTc intervals, and QRS complex (ms). The data are represented as means ± SEM. Data represent eight to nine mice per group. Significant differences between infected and uninfected groups *P < 0.05 (Mann–Whitney test). [file Image_1.tif]

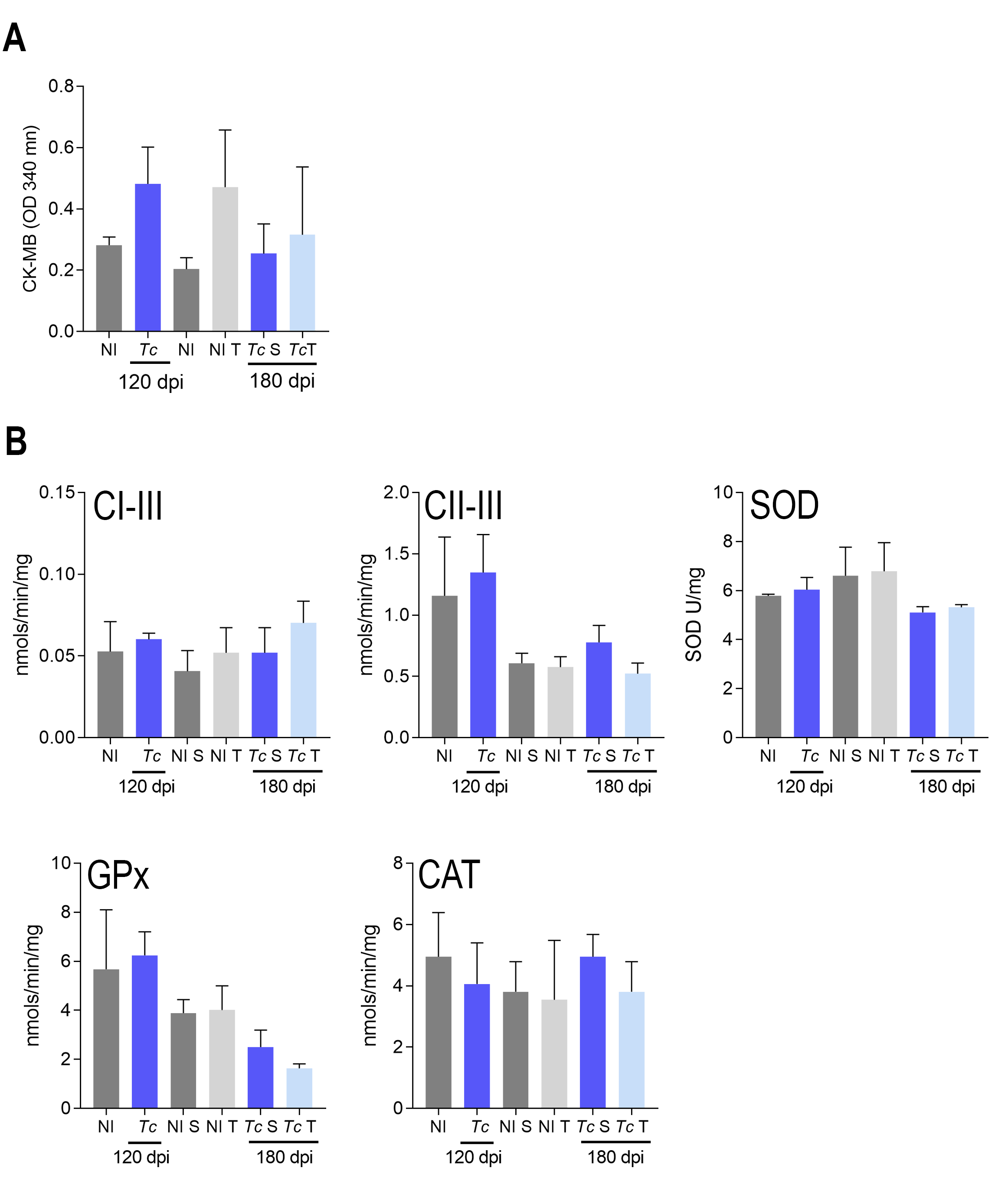

Supplement: Supplementary Figure S2 — Effect of T. cruzi infection and physical exercise on CK-MB, mitochondrial and antioxidant activities in C57BL/6 mice. (A) CKMB activity. (B) Mitochondrial complex and antioxidant activities. Data are represented as means ± SEM. Data represent six to eight mice per group (Mann–Whitney; Kruskal–Wallis test, Dunn’s posttest). [file Image_2.tif]
